# Geological control of floristic composition in Amazonian forests

**DOI:** 10.1111/j.1365-2699.2011.02585.x

**Published:** 2011-11

**Authors:** Mark A Higgins, Kalle Ruokolainen, Hanna Tuomisto, Nelly Llerena, Glenda Cardenas, Oliver L Phillips, Rodolfo Vásquez, Matti Räsänen

**Affiliations:** 1 University Program in Ecology, Nicholas School of the Environment, Duke UniversityDurham, NC 27701, USA; 2 Department of Biology, University of TurkuFI-20014 Turku, Finland; 3 Facultad de Ciencias del Ambiente y Biotecnología, Universidad Particular de IquitosIquitos, Peru; 4 Earth and Biosphere Institute, School of Geography, University of LeedsLeeds LS2 9JT, UK; 5 Proyecto Flora del Perú, Jardín Botánico de MissouriJaen, Cajamarca, Peru; 6 Department of Geology, University of TurkuFI-20014 Turku, Finland

**Keywords:** Amazonia, Andean uplift, edaphic gradients, floristic composition, geological formations, Landsat, Melastomataceae, pteridophytes, SRTM, vegetation mapping

## Abstract

**Aim:**

Conservation and land-use planning require accurate maps of patterns in species composition and an understanding of the factors that control them. Substantial doubt exists, however, about the existence and determinants of large-area floristic divisions in Amazonia. Here we ask whether Amazonian forests are partitioned into broad-scale floristic units on the basis of geological formations and their edaphic properties.

**Location:**

Western and central Amazonia.

**Methods:**

We used Landsat imagery and Shuttle Radar Topography Mission (SRTM) digital elevation data to identify a possible floristic and geological discontinuity of over 300 km in northern Peru. We then used plant inventories and soil sampling to document changes in species composition and soil properties across this boundary. Data were obtained from 138 sites distributed along more than 450 km of road and river. On the basis of our findings, we used broad-scale Landsat and SRTM mosaics to identify similar patterns across western and central Amazonia.

**Results:**

The discontinuity identified in Landsat and SRTM data corresponded to a 15-fold change in soil cation concentrations and an almost total change in plant species composition. This discontinuity appears to be caused by the widespread removal of cation-poor surface sediments by river incision to expose cation-rich sediments beneath. Examination of broad-scale Landsat and SRTM mosaics indicated that equivalent processes have generated a north–south discontinuity of over 1500 km in western Brazil. Due to similarities with our study area, we suggest that this discontinuity represents a chemical and ecological limit between western and central Amazonia.

**Main conclusions:**

Our findings suggest that Amazonian forests are partitioned into large-area units on the basis of geological formations and their edaphic properties. The evolution of these units through geological time may provide a general mechanism for biotic diversification in Amazonia. These compositional units, moreover, may correspond to broad-scale functional units. The existence of large-area compositional and functional units would suggest that protected-area, carbon sequestration, and other land-use strategies in Amazonia be implemented on a region-by-region basis. The methods described here can be used to map these patterns, and thus enable effective conservation and management of Amazonian forests.

## Introduction

Amazonian forests are globally important because of their role in nature conservation and climate regulation. Amazonia contains the largest remaining tracts of undisturbed tropical forest on Earth; harbours record numbers of plant and animal species; and stores and processes more biomass carbon than any other region (Gentry, 1988; Malhi *et al.*, 2008). Amazonian forests are also the object of substantial economic interest in the form of oil exploration, timber extraction, agribusiness and colonization (Soares *et al.*, 2006; Finer *et al.*, 2008). Reconciling conflicting interests in this area will require high-resolution maps of compositional and functional patterns, and an understanding of the factors that control them (Dinerstein *et al.*, 1995; Margules & Pressey, 2000).

Substantial doubt exists, however, about the existence and determinants of floristic divisions in Amazonian forests. The search for large-area divisions in Amazonia has generally focused on possible past or present dispersal barriers in these apparently uniform forests. The Pleistocene refugia hypothesis proposes that speciation in isolated forested refugia during periods of aridity and forest contraction resulted in broad-scale patterns in endemism and species distributions across Amazonia, as suggested by animal, soils and palynological data (Haffer, 1969; Absy *et al.*, 1991; Pessenda *et al.*, 2001). Large-area floristic units have consequently been delimited on the basis of postulated Pleistocene forest refugia (Prance, 1982, 1994). More recent studies, however, find no evidence of Pleistocene forest fragmentation in the Amazonian lowlands, suggesting that Pleistocene climate change has not been a primary driver of contemporary floristic patterns (Colinvaux & De Oliveira, 2001; Colinvaux *et al.*, 2001; Kastner & Goñi, 2003).

Amazonian forests have also been divided into large-area units on the basis of large rivers and their floodplains (Dinerstein *et al.*, 1995; Olson *et al.*, 2001), under the longstanding belief that these rivers serve as barriers to plant and animal movement (Wallace, 1852). Large rivers are believed to serve as dispersal boundaries for primate and avian taxa, and are commonly used as limits in avian range maps (Ayres & Cluttonbrock, 1992; Hayes & Sewlal, 2004; Schulenberg *et al.*, 2007; Pomara, 2009). The role of rivers in limiting other animal taxa is uncertain, however, and there is no evidence that rivers and their floodplains constitute important limits for plant species distributions (Patton *et al.*, 1994; Symula *et al.*, 2003; Pomara, 2009). In addition, it has been suggested that tectonic arches form dispersal barriers in Amazonian forests (Lougheed *et al.*, 1999; Patton *et al.*, 2000). These arches are largely subsurface structures, however, and elevation differences across them are usually insufficient to limit plant or animal movement (Rossetti *et al.*, 2005; Wesselingh & Salo, 2006).

An alternative candidate for floristic patterns in Amazonian forests is edaphic variation among geological formations. Broad-scale studies of Amazonian forests have found strong relationships between plant species composition and soil properties, suggesting that floristic pattern may be driven by underlying geological patterns (Tuomisto *et al.*, 1995, 2003c; ter Steege *et al.*, 2006). Higher-resolution studies in western Amazonia have found abrupt changes in plant species composition in terra firme (non-inundated) forest, corresponding to abrupt changes in soil properties and topography, further indicating a link between geological formations and floristic composition (Phillips *et al.*, 2003a; Tuomisto *et al.*, 2003b; Pitman *et al.*, 2008). This relationship has been difficult to test, however, due to a lack of floristic, edaphic and geological data for these vast and remote forests.

Here we ask whether Amazonian forests are partitioned into broad-scale floristic units on the basis of geological formations and their edaphic properties. Our study focuses on geological and edaphic patterns in western and central Amazonia, which are the result of three phases of deposition and erosion dating to the Miocene. First, early Andean uplift during the early to middle Miocene (*c*. 25–10 Ma) closed the then-prevailing north-western drainage of western Amazonia, resulting in the creation of the widespread Pebas Embayment and deposition of the Pebas Formation. The Pebas Formation (equivalent to the Brazilian Solimões Formation) is distributed across western and central Amazonia, and consists of poorly weathered and cation-rich clay sediments deposited under the low-energy semi-marine or lacustrine conditions of the Pebas Embayment [Räsänen *et al.*, 1995; Hoorn *et al.*, 2010; but see Latrubesse *et al.* (2010) for an alternative dating of the Pebas Formation]. Second, continued Andean uplift during the late Miocene (*c*. 10–5 Ma) caused the draining of the Pebas Embayment and the establishment of the modern eastern drainage of the Amazon River, and a transition to predominantly fluvial or deltaic deposition across western Amazonia (Figueiredo *et al.*, 2009; Hoorn *et al.*, 2010). Under these high-energy conditions, the Pebas Formation was largely overlain by sandy, weathered and cation-poor fluvial sediments, such as the Nauta Formation in Peru and the Içá Formation in central Amazonia (Schobbenhaus *et al.*, 2004; Rebata *et al.*, 2006). Third, ongoing Andean uplift during the Plio-Holocene (*c*. 5 Ma to recent) caused a transition across much of western Amazonia from deposition to fluvial erosion (Räsänen *et al.*, 1990). Under these increasingly high-energy conditions, river incision and denudation have removed the more friable and cation-poor late Miocene fluvial deposits across much of western Amazonia, and exposed vast expanses of the buried, cation-rich Pebas Formation (Räsänen *et al.*, 1990; INGEMMET, 2000; Roddaz *et al.*, 2005).

These processes have generated two types of geological and edaphic pattern in western and central Amazonia. First, due to the uneven nature of Plio-Holocene uplift and drainage incision, islands of cation-poor late Miocene fluvial deposits remain across western Amazonia, elevated above the cation-rich Miocene Pebas matrix (Räsänen *et al.*, 1990; INGEMMET, 2000). These include the Nauta Formation in northern Peru, and mesa-like islands of elevated, cation-poor soils along the Curaray, Napo and Putumayo rivers (INGEMMET, 2000; Tuomisto *et al.*, 2003b; Rebata *et al.*, 2006). Second, the original late Miocene depositional surface remains undisturbed in central Amazonia, where it is represented by the widespread late Miocene–Pleistocene Içá Formation (Schobbenhaus *et al.*, 2004; Rossetti *et al.*, 2005). Geological studies indicate that the Pebas and Içá Formations meet in western Brazil (Räsänen *et al.*, 1990; Schobbenhaus *et al.*, 2004), and this may represent an edaphic limit between western and central Amazonia (Sombroek, 1991).

To understand the importance of these geological patterns for Amazonian biota, we conducted an extensive field campaign guided by a combination of Landsat satellite imagery and Shuttle Radar Topography Mission (SRTM) digital elevation data. Previous studies in Amazonia have found that floristic patterns can be inferred from Landsat data (Tuomisto *et al.*, 1995, 2003a,b; Salovaara *et al.*, 2005), and geological patterns from SRTM data (Rossetti & Valeriano, 2007; Latrubesse *et al.*, 2010). Our strategy was to identify matched patterns in these data, suggesting floristic patterns of geological origin, and then use plant inventories and soil sampling to test this relationship. We then used Landsat and SRTM data to identify similar patterns in western and central Amazonia.

## Materials and methods

### Study area

To study the relationship between geological formations and plant species composition in Amazonia, we selected a study area of *c*. 250 × 350 km in northern Peru, centred on a documented boundary between two geological formations: the Miocene Pebas Formation in the north, and the late Miocene Nauta Formation in the south (INGEMMET, 2000; Rebata *et al.*, 2006). These formations are nearly horizontal, such that the Nauta Formation overlies the Pebas Formation in the south of our study area but is absent in the north. Access to the study area was provided by the Tigre, Pucacuro and Pastaza rivers, and by a road between the Pastaza and Tigre rivers that services an oil concession. In addition to the Pebas and Nauta Formations, our study area also contains portions of the Pastaza Fan in the south and west. The Pastaza Fan is a vast Holocene volcanoclastic alluvial fan deposited by the Pastaza River and draining the Cotopaxi volcano (Räsänen *et al.*, 1990, 1992). We concentrated our sampling on the Nauta and Pebas formations, and sampled only three locations in the Pastaza Fan.

The climate in the study area is tropical, humid and almost aseasonal. Mean monthly temperature in the city of Iquitos (250 km distant) is 25–27 °C throughout the year, and annual precipitation is about 3100 mm (Marengo, 1998). Elevation in the study area ranges from 98 to 433 m above sea level, and the landscape ranges from flat to precipitously hilly. Human population density in this area is low, and access by boat or airplane is highly limited. In addition, the impacts of oil concession activities upon vegetation are restricted to narrow clearings along roads and pipelines. As a consequence, anthropogenic disturbance in the study area is small, although some timber is extracted from forests along the Tigre River.

The vegetation in the study area consists primarily of closed-canopy, broadleaf evergreen rain forest typical of western and central Amazonia. The majority of this forest is non-inundated (terra firme), although seasonally or sporadically inundated zones are found along rivers and large streams, and the southernmost portion of the Pastaza Fan is inundated seasonally or permanently. We used both field observations and SRTM elevation data to ensure that our sampling was limited to undisturbed, terra firme forest of the geological formations of interest, and we excluded sites with alluvial soils in inundated areas.

We constructed Landsat and SRTM image mosaics for the study area, and sampled it for plants and soils in the field. We also constructed Landsat and SRTM mosaics for western and central Amazonia in order to examine other regions for similarities to our study area. The proposed interface of the Pebas and Içá Formations, an area of *c*. 600 × 900 km, was particularly interesting and was selected for further study. This area includes parts of western Brazil, eastern Colombia and eastern Peru, and the drainages of the Caquetá, Putumayo, Amazon and Juruá rivers.

### Remote sensing data

**Figure 1:**
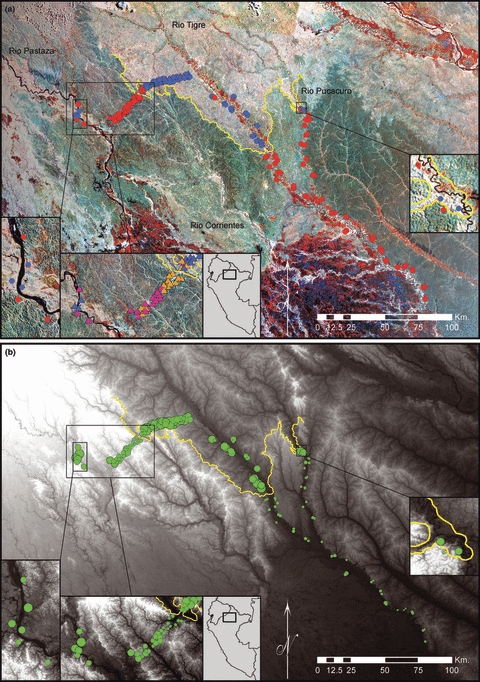
Relationship between remotely sensed discontinuity, plant species composition and soil cation concentrations at the study area in northern Peru. In both panels, the yellow line indicates the discontinuity identified in Landsat and Shuttle Radar Topography Mission (SRTM) data between the Miocene Pebas Formation (to the north) and late Miocene Nauta Formation (to the south). (a) Results of floristic cluster analyses superimposed upon a Landsat mosaic for the study area. Circles indicate transects at which both pteridophyte and Melastomataceae data were collected; triangles indicate transects at which only pteridophyte data were collected. The colour of a transect indicates its classification by cluster analysis into one of two groups (blue and red; main figure and far left and right insets), or one of four groups (blue, pink and orange; centre-left inset). For the two-group solution, pteridophyte and Melastomataceae analyses gave identical results, and the colour of the transects indicates the clustering result for both plant datasets. For the four-group solution, only clustering results for pteridophyte data are reported, and a group of three inventories restricted to the south-east of the study area is not displayed in the inset. Dark red tones along rivers and in the south of the study area indicate inundated forest or swamp; white patches in the north are clouds. (b) Soil cation concentrations superimposed upon an SRTM digital elevation model for the study area. The diameter of circles is proportional to the log-transformed sum of the concentrations of four cations (Ca, Mg, Na and K). Light tones in the digital elevation model indicate higher elevation (maximum 433 m) and dark tones lower elevation (minimum 98 m). From left to right, insets show detail of the Pastaza Fan; detail of north-west of study area; location of study area; and detail of the upper Pucacuro River.

**Figure 2:**
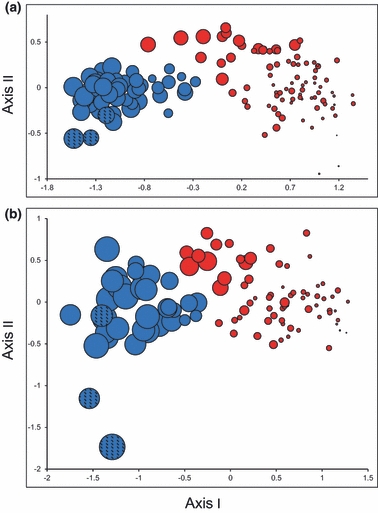
Relationship between plant species composition, cluster analysis groups and soil properties for the study area in northern Peru. Plot represents the first two non-metric multidimensional scaling axes for (a) pteridophytes (138 transects); (b) Melastomataceae (104 transects). Points in the plot are sized by the log-transformed sum of the concentrations of four cations (Ca, Mg, Na and K) and coloured according to the two-group cluster analysis solution in Fig. 1a. Striped circles indicate the three transects located in the Pastaza Fan. Axes I and II explain (a) 85 and 6%, respectively, of the variation for pteridophytes; (b) 69 and 9%, respectively, of the variation for Melastomataceae.

We used Landsat satellite image mosaics to identify possible floristic patterns in our field study area and our broader area of interest. We acquired orthorectified Landsat Geocover imagery (Tucker *et al.*, 2004) from the Global Landcover Facility (GLCF, University of Maryland, USA, http://glcf.umiacs.umd.edu). Geocover imagery is free to the public and orthorectified to high positional accuracy (< 50 m root mean square error, RMSE). In total we used 11 images for our study site in northern Peru, and 29 images for our area of interest in western Brazil (Appendices S1 and S2 in Supporting Information). We constructed image mosaics using bands four, five and seven of the Landsat images (Appendix S1), as these bands have been found to represent floristic patterns (Tuomisto *et al.*, 2003a). All image mosaicing was conducted in Erdas Imagine v. 8.7 (ERDAS Inc., Atlanta, GA, USA). For display and interpretation, we assigned bands four, five and seven to red, green and blue, respectively. Image interpretation was performed manually by M.H. (Appendix S1), yielding the boundaries observed in Figs 1 & 2, but simple automated classification techniques (principal components analysis and image thresholding) give similar results. Interpretations are conservative and the actual extent of the boundaries may be longer than indicated.

We used SRTM digital elevation data (Rodriguez *et al.*, 2006) to identify geological formations. SRTM data were downloaded from the USGS National Map Seamless Server (http://seamless.usgs.gov) in 83 non-overlapping tiles. These data are free to the public and are of high positional and elevational accuracy (9 and 6 m, respectively). These were then mosaiced into a single image in ArcGIS v. 9.1 that encompasses all of South America north of Chile (*c*. 20° S). Contrast stretches were applied uniformly to maps and insets to improve interpretability. Image interpretation was performed by M.H. and M.R. with the objective of identifying geomorphological features and geological processes. To quantify changes in elevation across the boundaries identified in our Landsat and SRTM data, we used ArcGIS v. 9.1 to construct 10 km buffers on either side of these boundaries and calculate mean elevation within these buffers. These values were then used to compute the mean differences in elevation across the boundaries. Because SRTM data reflect the elevation of the forest canopy rather than the ground, these data thus somewhat overestimate absolute elevations (http://www2.jpl.nasa.gov/srtm/faq.html). Because our study area is covered by uniform, closed-canopy forest, however, differences in elevation should primarily reflect topography of the terrain.

### Plant and soil sampling

We required a field dataset that was sufficiently extensive to sample the width of large geological formations, but also sufficiently dense to detect abrupt changes in soil properties and floristic composition. To avoid the sampling problems associated with trees – including large numbers of stems and taxa, tall height and taxonomic uncertainty – we focused our field data collection on pteridophytes (ferns and lycophytes) and the Melastomataceae (a family of shrubs and small trees). These plant groups have been found to reproduce the majority of the floristic pattern observed in tree inventories with a much smaller time investment (Tuomisto *et al.*, 1995; Ruokolainen *et al.*, 1997, 2007), consistent with other findings for taxa-based inventory (Higgins & Ruokolainen, 2004).

We sampled plants and soils at 138 sites using a standard procedure (Tuomisto *et al.*, 2003a,c;). Sampling sites were located so as to represent the range of variation observed in the Landsat imagery for non-inundated areas. At each site we established a 5 × 500 m linear transect, along which we collected presence–absence data for pteridophytes. At a subset of 104 sites we also collected presence–absence data for the Melastomataceae. For pteridophytes, only individuals with at least one leaf longer than 10 cm were recorded, and epiphytic and climbing individuals were included only if they had green leaves at a height ≤ 2 m above ground. For Melastomataceae, all individuals except seedlings smaller than 5 cm long were recorded. Voucher collections for all species were deposited in herbaria in Peru (AMAZ and USM) and Finland (TUR) (herbarium acronyms according to *Index herbariorum*; Thiers, nd).

We also collected soil samples at three locations along each transect (at 50, 250 and 450 m from the start of the transect). Each soil sample consisted of five subsamples of the top 10 cm of mineral soil, collected in an area of *c*. 4 × 4 m, and these five subsamples were pooled into a single sample in the field. Soil samples were then analysed at MMT Agrifood (Jokioinen, Finland) for pH; loss on ignition (a measure of organic matter content); P concentration (Bray method); and extractable Al, Ca, K, Mg and Na. In addition, percentages of sand, silt and clay were determined at the University of Turku (Finland). For the first 57 transects, the three samples for each transect were analysed separately and their mean value was used in numerical analyses. For the remaining 87 transects, equal dry weights of the three soil samples were combined and analysed as a single sample.

To estimate the importance of the edaphic variation in our study area for tree species composition, we used previously reported tree plot data from a neighbouring inventory network (Phillips & Miller, 2002; Phillips *et al.*, 2003b). From this network, we used all plots that were situated in terra firme forests and for which standardized abundance data and soil data were available, for a total of 14 plots (Allpgen1, Allpihan, Allpisac, Allp4, CN-01, CN-02, CN-03, CS-01, CS-02, CS-03, IN-01, SU-01, Yanamono 1 and Yanamono 2). Each 0.1 ha plot consisted of ten 2 × 50 m subplots in which all tree stems with diameter at breast height ≥ 2.5 cm were identified to species or morphospecies. Lianas and stranglers were excluded from our analysis. Further information on these plots – including location, species richness and number of individuals – can be found in Phillips *et al.* (2003b) and references therein. Soil samples from the tree plots were analysed at the same laboratory as those from our transects, and the soil data for these two datasets are thus directly comparable.

### Data analyses

We used hierarchical agglomerative cluster analysis (unweighted pair-group method using arithmetic averages, UPGMA) to identify compositional patterns in our plant datasets (Legendre & Legendre, 1998; Tuomisto *et al.*, 2003b). Cluster analysis uses pairwise compositional distances between sites to group them based on their species composition, and these groups are indicative of vegetation types. We quantified pairwise compositional dissimilarities between sites using the one-complement of the Jaccard similarity index. This dissimilarity measure expresses the number of species unique to either of the sites as a proportion (or percentage if multiplied by 100) of the total number of species in the two sites. These calculations were performed with pc-ord v. 4.41 (MjM Software, Gleneden Beach, OR, USA).

We used indicator species analysis to test whether the distribution patterns of individual plant species matched the two main groups generated by the cluster analysis (Dufrêne & Legendre, 1997). We calculated indicator values for all pteridophyte and Melastomataceae species present at seven or more sites (the minimum frequency required to return a significant value in the indicator species analysis) for the two-group clustering solution, and calculated the statistical significance of each indicator value using 999 permutations. Using these results, we calculated the percentage of species that were significantly associated with each cluster analysis group. These calculations were performed with IndVal v. 2.0 (available at http://www.bio.umontreal.ca/Casgrain/en/labo/index.html).

We used non-metric multidimensional scaling (NMDS) to visualize the relationship in our study area between plant species composition, cluster analysis groups and soil properties. We calculated two-dimensional NMDS solutions for both our pteridophyte and Melastomataceae data, using the one-complement of the Jaccard index as a distance measure, running a maximum of 400 iterations from 40 random starting configurations, and applying an instability criterion of 10^−5^. We then overlaid these ordination results with clustering results and information on soil properties. NMDS calculations were performed with pc-ord v. 4.41.

We used multiple regression on distance matrices (Legendre *et al.*, 1994; Phillips *et al.*, 2003a; Tuomisto *et al.*, 2003a,c;) to assess the relationships between floristic dissimilarities (dependent variables), and edaphic and geographical dissimilarities (independent variables). For these analyses, we used three groups of independent variables: edaphic distances only, geographic distances only, and edaphic plus geographic distances. This allowed us to quantify the shared and unique contributions of edaphic and geographic distances to explaining the total variation in floristic distances (Tuomisto *et al.*, 2003c; Ruokolainen *et al.*, 2007). We ran regression analyses separately for pteridophytes and Melastomataceae, and measured compositional dissimilarity using the one-complement of the Jaccard index in both cases. The independent edaphic variables were distance matrices based on pH; loss on ignition (LOI); log-transformed concentrations of P, Al, Ca, K, Mg and Na; and percentages of sand, silt and clay. The geographical distance matrix was log-transformed before analysis. We used backwards elimination to exclude from the final regression model those distance matrices whose contribution to explaining the variation in the floristic distances was statistically non-significant (*P*≥0.1 after Bonferroni correction). These calculations were performed with Permute! v. 3.4 (http://www.bio.umontreal.ca/Casgrain/en/labo/index.html).

Finally, we divided the tree plots into two groups, such that the ranges of soil cation content in each group matched those on either side of the boundary in our study area between the Pebas and Nauta formations. We then calculated indicator species values for all tree species present in five or more plots, as described above. Abundance data were used for these analyses, and results were calculated either for all tree species, or only for the most abundant species (species with an average density of at least one individual per plot).

## Results

### Satellite image interpretation: Pebas–Nauta boundary

Inspection of the Landsat mosaic for our Peruvian study area revealed a discontinuity of over 300 km between a region of light tones in the north of our study area and a region of dark tones in the south (Fig. 1a). These regions correspond to the Pebas Formation and Nauta Formation, respectively (Appendix S3a), and this difference in tone suggests a difference in plant species composition between the two formations. Coincident with this discontinuity, we observed a 20 m topographic discontinuity in SRTM data between a lower-elevation surface to the north of the boundary, corresponding to the Pebas Formation; and a higher-elevation surface to the south, corresponding to the overlying Nauta Formation (Fig. 1b). Overlaid upon this local change in elevation is a broad tilt in the study area from higher elevations in the north-west to lower elevations in the south-east, indicative of Andean uplift.

On the basis of these data, we propose that Andean uplift in the north along the Vaupes and Iquitos arches has caused the upper reaches of the Tigre and Pucacuro rivers to incise through and remove the more friable sediments of the Nauta Formation and expose the older Pebas Formation sediments beneath (INGEMMET, 2000; Roddaz *et al.*, 2005). This sediment removal is occurring along two v-shaped erosion frontiers that are progressing from north-west to south-east along the courses of these two rivers (Fig. 1, yellow line). These findings suggest that the floristic patterns indicated by the Landsat data were caused by the removal of the Nauta Formation to expose the Pebas Formation beneath.

Two other features are apparent in our study area. First, the Pastaza Fan is represented in our Landsat mosaic by dark red and light pink tones in the south and west of the study area, respectively. The dark red tones in the south correspond to the inundated and actively aggrading portions of the Fan, whereas the light pink tones in the west correspond to the non-inundated and inactive portions of the Fan. Due to the relatively recent Pleistocene deposition of the Pastaza Fan (Räsänen *et al.*, 1992), it is not dissected by rivers and appears as a smooth but tilted surface in the SRTM data. Second, dark red tones along rivers in our study area correspond to floodplain forests, and these appear as smooth, lower-elevation surfaces in the SRTM data. Inundated forest is already known to be compositionally distinct from terra firme forest, and thus was not sampled in this study (as described above).

### Floristic and edaphic patterns at the Pebas–Nauta boundary

We inventoried plants and collected soils at 138 sites along more than 450 km of road and river. In total, we encountered 191 pteridophyte species (average 31 per transect) and 210 Melastomataceae species (average 32 per transect).

The boundary between the Pebas and Nauta Formations corresponded to an abrupt and profound discontinuity in plant species composition. Separate cluster analyses of Pteridophyte and Melastomataceae data grouped the sites identically into two groups, almost perfectly separated by the discontinuity and corresponding to the two geological formations (Fig. 1a, groups coloured red and blue). The average compositional difference between transects from different cluster analysis groups was 89% for both pteridophytes and Melastomataceae (one-complement of the Jaccard index, expressed as a percentage), and *c*. 80% of both pteridophyte and Melastomataceae species were statistically significant indicators of one of the two groups (indicator species analysis, *P*<0.05; Appendix S4).

Neither of the two cluster analysis groups was internally homogeneous, however, and the average compositional difference among transects from the same group was 61 and 73% for pteridophytes and Melastomataceae, respectively. To study this internal variation, we used cluster analysis of our pteridophyte data to group the sites into four groups (results for the Melastomataceae are not reported due to substantially lower sampling in the north-west of the study area). The group corresponding to the Pebas formation remained unchanged, but the group corresponding to the Nauta Formation was shown to consist of three spatially distinct subgroups: a group of 18 sites on more cation-rich soils in the north-west of the Nauta Formation; a group of sites covering the majority of the Nauta Formation; and a group of three sites on exceptionally cation-poor soils in the south-east of the Nauta Formation. The distribution of the first and second of these groups along the Pastaza–Tigre road is shown in an inset of Fig. 1a (centre-left inset, groups coloured pink and orange, respectively), but the third group is not displayed. In addition, three of our westernmost transects were located in a branch of the Pastaza Fan (Fig. 1a, left inset). These sites were floristically distinct from neighbouring sites on the Nauta Formation, and were instead classified with the Pebas Formation sites.

The boundary between the two geological formations also corresponded to an abrupt and substantial change in soil properties (Fig. 1b). Sites in the Pebas Formation, to the north of the boundary, had on average 15 times greater soil cation concentrations than sites in the Nauta Formation to the south (sum of Ca, Na, Mg and K; Kruskal–Wallis test, *P*<0.0001). The three Pastaza Fan sites were excluded from this comparison, and their cation concentrations were similar to those on the Pebas Formation (Fig. 1b). Overall, cation concentrations in our study area ranged from 0.08 to 24.1 cmol(+) kg^−1^, comparable with the overall range documented for Brazilian and Peruvian Amazonia (< 0.1–39 cmol(+) kg^−1^; Sanchez & Buol, 1974; Botschek *et al.*, 1996).

NMDS ordinations for our pteridophyte and Melastomataceae data reveal a clear compositional gradient along the first NMDS axis, and the groups identified by our cluster analyses are cleanly separated along this axis (Fig. 2). Both this axis and the cluster analysis groups are strongly associated with soil cation concentrations: the Pearson correlation between the first NMDS axis and the log-transformed sum of bases (Ca, Na, Mg and K) is 0.95 for pteridophytes and 0.94 for Melastomataceae.

Using multiple regression on distance matrices, we found that a model including both edaphic dissimilarities and geographic distance explained 71 and 66% of the total variation in compositional dissimilarities for pteridophytes and Melastomataceae, respectively. Of this, edaphic distances uniquely explained 58 and 39%; geographic distance uniquely explained 2 and 7%; and the remaining 11 and 20% were jointly explained by edaphic and geographic dissimilarities. All of our edaphic models retained percentage silt and log-transformed concentrations of Al, Ca and Mg. The edaphic model for pteridophytes additionally retained pH and log-transformed K; and the model for Melastomataceae retained LOI. These results suggest that the floristic patterns in our study area are associated primarily with edaphic variation rather than dispersal dynamics or chance effects (Hubbell, 2001).

To test the importance of the edaphic discontinuity observed in our study area for tree species, we divided the 14 tree plots into two groups using a cation concentration of 2.09 cmol(+) kg^−1^ (sum of the concentrations of Ca, Mg, Na and K), the value that divided the pteridophyte transects into two groups with the smallest number of misclassifications relative to the floristic cluster analysis groups. This yielded two groups of seven tree plots each. Of the 65 tree species analysed, 28% were significantly associated with one of the soil-defined groups; and of the 29 most abundant tree species, 48% were significantly associated (indicator species analysis, *P*<0.05; Appendix S4). These findings are consistent with strong associations between tree species composition, soil variables and geological formation observed over very short distances in southern Peru (Phillips *et al.*, 2003a); and abrupt changes in tree species composition observed at sites close to ours (Pitman *et al.*, 2008).

These results, however, are conservative estimates of the tree species turnover expected along the edaphic gradient. As documented by Jones *et al.* (2008), the low individuals/species ratio for tree data makes it more difficult to obtain significant results. Furthermore, we lacked tree plot data for the top quarter of the cation gradient in our study area. To estimate the effect of this shorter cation gradient, we used a subset of our transects that corresponded to the truncated cation gradient, divided them into two groups using the 2.09 cmol(+) kg^−1^ cutoff, and repeated the indicator analyses for pteridophytes and Melastomataceae. The percentage of species showing significant associations with the two cation classes fell from 87% in the full dataset to 71% in the truncated dataset for pteridophytes, and from 76 to 61% for Melastomataceae (for species present in eight or more transects; *P*<0.05; Appendix S4). Including the top quarter of the cation gradient thus increased the number of significantly associated species by about 25% in these plant groups, and we expect that a comparable increase would be observed with trees.

### Satellite image interpretation: Pebas–Içá boundary

**Figure 3:**
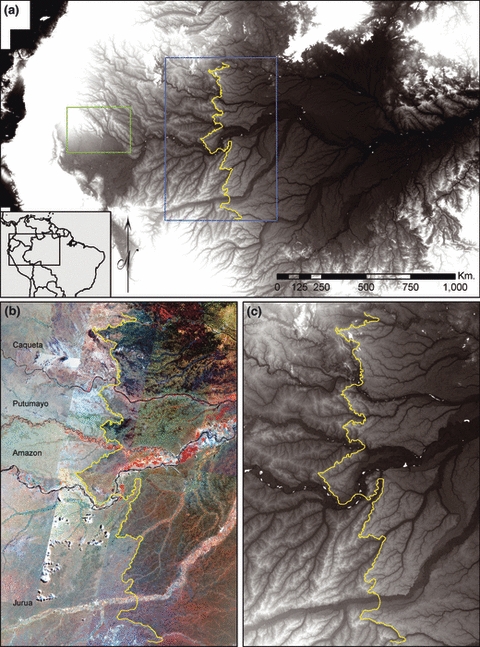
Boundary between Miocene and late Miocene–Pleistocene sediments relative to continent-scale Shuttle Radar Topography Mission (SRTM) and Landsat mosaics. In all panels, the yellow line indicates the boundary between the dissected Miocene Pebas Formation in the west and the planar late Miocene–Pleistocene Içá Formation in the east. (a) SRTM digital elevation model for Amazonia. Light tones indicate higher elevation (maximum 6157 m) and dark tones lower elevation (minimum 0 m). Green box indicates the extent of Fig. 1; blue box indicates the extent of panels (b) and (c); inset indicates the location of the figure relative to northern South America. (b) Landsat mosaic with major rivers labelled. (c) SRTM digital elevation model (elevations range from 40 to 779 m).

To determine whether similar floristic discontinuities exist elsewhere in Amazonia, we examined Landsat and SRTM mosaics for western and central Amazonia. We found a north–south discontinuity of at least 1500 km in western Brazil (Fig. 3b), between a vast area of light tones in the west of the Landsat mosaic, and a similarly large area of dark tones in the east. These areas correspond approximately to the Pebas and Içá Formations, respectively (Schobbenhaus *et al.*, 2004; Appendix S3b), and this difference in tone suggests a widespread difference in floristic composition between the two formations. Inspection of the SRTM mosaic revealed a matching discontinuity between a dissected, lower-elevation surface in the west of the mosaic, corresponding to the Pebas Formation; and a planar, higher-elevation surface in the east, corresponding to the Içá Formation (Fig. 3a,c). The Içá Formation is elevated *c*. 21 m above the underlying Pebas Formation, remarkably consistent with the difference in elevation of 20 m between the Nauta and Pebas Formations observed in our Peruvian study area. This suggests, as in our Peruvian study area, that erosion to the west of the boundary has removed the overlying late Miocene–Pleistocene sediments of the Içá Formation and exposed the Miocene Pebas Formation beneath, resulting in an abrupt and profound change in soil chemistry.

In addition, the erosional processes responsible for the Pebas–Içá boundary appear identical to those responsible for the Pebas–Nauta boundary. As in our Peruvian study area (Fig. 1), the Pebas–Içá discontinuity (Fig. 3) consists of a series of v-shaped erosion frontiers culminating at the channels of the Juruá, Amazon, Putumayo and Caquetá rivers and their tributaries. We propose that these erosion frontiers are driven by uplift at the Serra do Moa, Iquitos and Vaupes arches, west of the boundary, and are progressing eastwards along the courses of these rivers. The net effect is an eastwards-advancing front of erosion of at least 1500 km, separating the incised and recently exposed Pebas Formation in the west from the undisturbed and planar late Miocene–Pleistocene depositional surface in the east. This boundary is *c*. 1000 km from the Andean orogeny, indicating long-distance influence of the Andean uplift upon lowland Amazonian forests.

## Discussion

Our results suggest that Amazonian forests are partitioned into discrete, large-area units on the basis of geological formations and their edaphic properties. In agreement with previous studies, we find evidence for floristic discontinuities in Amazonian forests (Tuomisto *et al.*, 2003b; Pitman *et al.*, 2008). We further demonstrate that these discontinuities (1) can extend for hundreds or possibly thousands of kilometres; (2) correlate with abrupt changes in soil properties; and (3) are driven by underlying geological discontinuities. Given the distances of the discontinuities observed here from the Andean orogeny, we conclude that Andean uplift is acting over distances of hundreds to thousands of kilometres to control floristic composition in Amazonia.

Our results also demonstrate that the combination of Landsat imagery and SRTM data is a powerful tool for identifying geological and floristic boundaries in Amazonia. Using matching patterns in these data, we identified a discontinuity of over 300 km in western Amazonia corresponding to a 15-fold change in cation concentrations and an almost total change in plant species composition for the taxa sampled here. On the basis of geological maps and additional field data, we believe these patterns are representative of widespread island-matrix organization across north-western Amazonia (INGEMMET, 2000; Higgins, 2010).

Using Landsat and SRTM data, we also identified a putative geological and floristic discontinuity of over 1500 km in western Brazil. Based on strong similarities with our Peruvian study area, we believe that similar geological processes are operating in western Brazil, and have yielded equally substantial differences in soils and floristic composition. Although we lack field data for this discontinuity, it corresponds to abrupt changes in mammal genotypes (Patton *et al.*, 1994; da Silva & Patton, 1998), and fish species composition and biomass (Arbeláez *et al.*, 2008), along the Jurua and Amazon rivers, respectively. It also coincides with large-area changes in soil properties (Sombroek, 1991) and forest physiognomy (IBGE, 2004; Appendix S3c), and corresponds approximately to the boundary between the widespread Pebas and Içá Formations (Räsänen *et al.*, 1990; Schobbenhaus *et al.*, 2004; Appendix S3b). We propose that this discontinuity represents a chemical and ecological limit between western and central Amazonia, and recommend that geological, soil and biological sampling be targeted at this vast boundary.

These two discontinuities are particularly interesting for three reasons. First, Amazonian forests have typically been divided into large-area units on the basis of large rivers and their floodplains (Dinerstein *et al.*, 1995). Our findings, however, reveal the existence of floristic discontinuities of hundreds or possibly thousands of kilometres in terra firme forest, indicated in the field only by changes in topography. These boundaries, furthermore, do not follow rivers, instead running perpendicular to them. This suggests that existing maps may not accurately describe floristic patterns in Amazonia, and may need to be revised to better enable conservation planning and management.

Second, our results contradict the commonly held view that broad-scale floristic patterns in western Amazonia reflect a gradual transition from recently deposited and nutrient-rich sediments of Andean origin in the west to older, weathered and nutrient-poor soils in the east (Irion, 1984; Terborgh & Andresen, 1998; ter Steege *et al.*, 2006; Pitman *et al.*, 2008). The Pebas Formation is millions of years older than the overlying Nauta or Içá Formations, but has cation concentrations that are an order of magnitude greater. The presence of cation-rich Pebas sediments in western Amazonia, furthermore, is explained not by recent deposition, but rather by widespread removal of the cation-poor Nauta sediments that previously covered them (Räsänen *et al.*, 1990). Although we do find evidence for recent (i.e. Pleisto-Holocene) deposition of young, rich soils in the Andean foreland (i.e. the Pastaza Fan), these Pleisto-Holocene deposits do not form an unbroken belt of rich sediments along the Andean foreland, as previously suspected (Pitman *et al.*, 2008). Instead, cation-rich terra firme soils in north-western Amazonia are derived largely from the ancient Pebas Formation (INGEMMET, 2000; Schobbenhaus *et al.*, 2004). Edaphic and floristic patterns in Amazonia thus reflect not a simple east–west age gradient, but rather a complex history of deposition and erosion dating to the Miocene. This emphasizes the importance of integrating geological knowledge into ecological and evolutionary studies of these forests (Räsänen *et al.*, 1995; Tuomisto *et al.*, 1995; Hoorn *et al.*, 2010).

Third, in agreement with earlier studies, our results demonstrate that abrupt changes across floristic discontinuities can make a greater contribution to compositional patterns in western Amazonia than gradual changes over large distances (Phillips *et al.*, 2003a; Tuomisto *et al*., 2003a–c; Ruokolainen *et al.*, 2007; Pitman *et al.*, 2008). Our findings, however, do not preclude gradual changes in forest composition at broader spatial scales in response to biogeographic differentiation or variations in climate (Terborgh & Andresen, 1998; Tuomisto *et al.*, 2003c; ter Steege *et al.*, 2006). We thus suggest that geological formations provide a discrete edaphic framework upon which gradual variations due to climate, dispersal limitation and other factors are superimposed. This suggests that using climate alone to map current or future distributions of Amazonian species may yield erroneous results, and that these maps would benefit greatly by incorporating geological and edaphic data.

We also believe that these findings have implications for the function and evolution of Amazonian forests. Soil properties and floristic composition are correlated with ecosystem properties such as wood density, productivity and forest dynamics (Malhi *et al.*, 2004; Phillips *et al.*, 2004; ter Steege *et al.*, 2006). This suggests that the geological and compositional units described here may correspond to large-area functional units. Large-area functional patterns in Amazonia have typically been modelled using interpolation approaches that assume gradual change in ecosystem properties (Malhi *et al.*, 2006). Our results suggest that incorporating geological and floristic discontinuities into these models would yield substantially more accurate estimates of large-area patterns in Amazonian forest function.

Furthermore, contemporary geological and edaphic patterns in western Amazonia reflect a gradual transformation since the Miocene, from a landscape dominated by nutrient-poor late Miocene fluvial or deltaic deposits to one dominated by nutrient-rich Miocene Pebas Formation deposits. This landscape evolution may have provided a mechanism for species diversification in western Amazonia (Räsänen *et al.*, 1995; Hoorn *et al.*, 2010). Plant taxa that were adapted to the poor soils that prevailed in the late Miocene may have been triggered to radiate across nutrient-rich Pebas sediments as these became increasingly available through erosion during the Pleistocene. In addition, the poor-soil parent taxa may then have become confined to increasingly small islands of cation-poor sediments, isolated from the undisturbed expanses of poor soils in central Amazonia.

This scenario is consistent with the rapid Pleistocene diversification of the rich-soil plant genus *Inga* in western Amazonia (Richardson *et al.*, 2001), and the mid-Miocene–Pleistocene evolution in western Amazonia of clay-associated Burseraceae from species associated with nutrient-poor alluvial deposits (Fine *et al.*, 2005). This is also consistent with the Plio-Pleistocene diversification of primate and mammal taxa in western Amazonia (Lavergne *et al.*, 2010; de Thoisy *et al.*, 2010), and the recent divergence of western and eastern Amazonian avian (Aleixo & Rossetti, 2007; Ribas *et al.*, 2009), primate (Silva & Oren, 1996) and mammalian (Patton *et al.*, 1994; da Silva & Patton, 1998) taxa. Furthermore, avian taxa associated with patches of poor soil in western Amazonia show long-distance affinities with common species in central Amazonia (Alonso & Whitney, 2003). The scenario described above emphasizes the importance of integrating geological knowledge into evolutionary studies, and provides a spatially explicit and testable hypothesis for the diversification of the western Amazonian biota.

The existence of compositionally and functionally distinct regions in Amazonia would suggest that protected-area and carbon-sequestration strategies should be developed and implemented in a region-by-region manner. Our findings thus support the ecoregion-based conservation strategies of the World Wildlife Fund (WWF), the Nature Conservancy and other international conservation organizations (Redford *et al.*, 2003). Indeed, the WWF ecoregion map captures a substantial portion of the long boundary that we describe here (Dinerstein *et al.*, 1995; Olson *et al.*, 2001), probably due to its use of the IBGE vegetation map (IBGE 2004; Appendix S3c). However, because of this reliance on existing map data and the lack of accurate information about these forests, ecoregion delineations for Amazonia are known to be in need of revision to better address conservation needs (Dinerstein *et al.*, 1995).

To map ecological regions in Amazonia, we thus recommend a three-step strategy: (1) the use of Landsat and SRTM data to identify matched geological and floristic boundaries; (2) the use of rapid, taxa-based plant inventory, soil sampling and geological study to verify these boundaries; and (3) the use of satellite imagery and field data to map these boundaries and assess the accuracy of the resulting maps. Further sampling with additional plant or animal groups can then be used to determine the broader utility of the maps. These maps can then be used to create more effective strategies for the protection and use of the world's most extensive tropical forests.

## Biosketch

Our research group consists of scientists from Duke University (USA), the University of Turku (Finland), the Universidad Particular de Iquitos (Peru), the University of Leeds (UK), and the Missouri Botanic Gardens (USA). We have broad research interests, including large-area compositional and functional patterns in Amazonian forests, the influence of climate change upon forest composition and function, and the ecology and taxonomy of Amazonian plants.

Author contributions: M.H., H.T. and G.C. collected pteridophyte field data; K.R. and N.L. collected Melastomataceae field data; M.H., K.R., H.T., N.L. and G.C. collected study area soil samples; O.P. and R.V. collected tree plot field data and corresponding soil samples; M.H. assembled and interpreted Landsat and SRTM mosaics. M.H. and K.R. performed statistical analyses; M.H. and M.R. performed geological interpretation of SRTM data; and M.H., K.R., H.T., O.P. and M.R. wrote the paper.

## SUPPORTING INFORMATION

**Appendix S1** Construction, processing and interpretation of Landsat mosaics.

**Appendix S2** Landsat data used for (a) Fig. 1; (b) Fig. 3.

**Appendix S3** Boundary between (a) Pebas and Nauta Formations relative to boundary identified in Fig. 1; (b) Pebas and Içá Formations relative to boundary identified in Fig. 3; (c) IBGE vegetation types relative to boundary identified in Fig. 3.

**Appendix S4** Indicator species analysis results for (a) pteridophyte species; (b) Melastomatacae species; (c) tree species.

As a service to our authors and readers, this journal provides supporting information supplied by the authors. Such materials are peer-reviewed and may be re-organized for online delivery, but are not copy-edited or typeset. Technical support issues arising from supporting information (other than missing files) should be addressed to the authors.
