# Supplementary material for: Geological control of floristic composition in Amazonian forests
Source: J Biogeogr. 2011 Nov;38(11):2136–49. doi: 10.1111/j.1365-2699.2011.02585.x (PMC3253337; doi:10.1111/j.1365-2699.2011.02585.x)

**SUPPORTING INFORMATION**

**Geological control of floristic composition in Amazonian forests**

Mark A. Higgins, Kalle Ruokolainen, Hanna Tuomisto, Nelly Llerena, Glenda Cardenas, Oliver L. Phillips, Rodolfo Vásquez and Matti Räsänen

*Journal of Biogeography*

**Appendix S1. Construction, processing, and interpretation of Landsat mosaics**

Two to three Geocover images are available, globally, for each path/row combination, and we selected the image that was either least cloudy or of the same date as neighbouring images (Appendix S2; imagery available from http://glcf.umiacs.umd.edu). The only exception was the image used for path 8, row 62 (Appendix S2a), for which cloud-free Geocover imagery was not available. In this case we used an unrectified but cloud-free image, and orthorectified it using a Geocover image and SRTM data for that location.

All images were first subset to the extent of the final mosaic area and then assembled sequentially to produce the final mosaic. Dense clouds in image overlap areas were excluded from image matching when doing so improved the matching product (three cases: paths, rows 4,63; 4,64; 5,61), or were removed from images prior to mosaicking (one case: path 3, row 63).

Once assembled, our mosaics were processed uniformly to improve interpretability by contrast stretching and low-pass spatial convolution (i.e. smoothing), as recommended by Hill & Foody (1994) and Tuomisto *et al*. (1994), also using Erdas Imagine. We subsequently used ArcGIS v. 9.1 (ESRI Inc.) to remove linear brightness trends from each of the final mosaic bands in order to remove the brightness trend artifact noted by Toivonen *et al*. (2006).

For image interpretation, we used tone and texture to identify the primary terra-firme vegetation types in the mosaics and to delineate the boundaries between them. Tone was a function of the bands and their colour assignments, and we assigned Landsat bands four, five, and seven to red, green, and blue, respectively. In both mosaics, forests growing on the Pebas Formation were characterized by light-blue or light-red tones, and a uniformly smooth texture. Forests growing on the Nauta and Içá Formations were characterized by dark-olive or dark-blue tones, and Nauta Formation forests were additionally characterized by a hilly and shadowed texture. The two types were generally easy to distinguish, but their boundaries were less obvious along the upper Pucacuro in our Peruvian study area; and along the Caquetá in western Brazil. In all cases, image interpretation was conducted without reference to either field data or to existing geological and vegetation maps.

**REFERENCES**

Hill, R.A. & Foody, G.M. (1994) Separability of tropical rain-forest types in the Tambopata-Candamo Reserved Zone, Peru. *International Journal of Remote Sensing*, **15**, 2687-2693.

Toivonen, T., Kalliola, R., Ruokolainen, K. & Malik, R.N. (2006) Across-path DN gradient in Landsat TM imagery of Amazonian forests: a challenge for image interpretation and mosaicking. *Remote Sensing of Environment*, **100**, 550-562.

Tuomisto, H., Linna, A. & Kalliola, R. (1994) Use of digitally processed satellite images in studies of tropical rain forest vegetation. *International Journal of Remote Sensing*, **15**, 1595-1610.


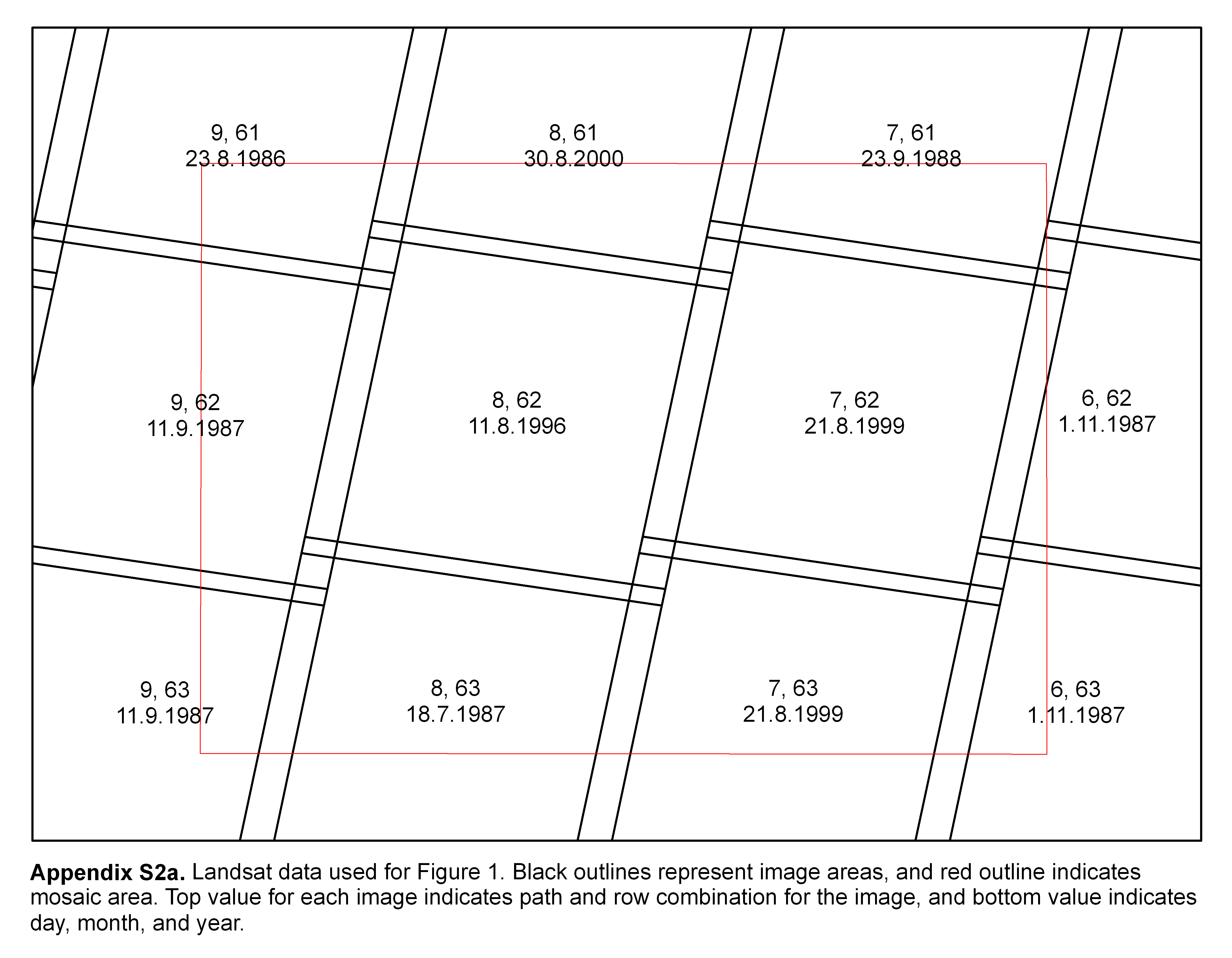


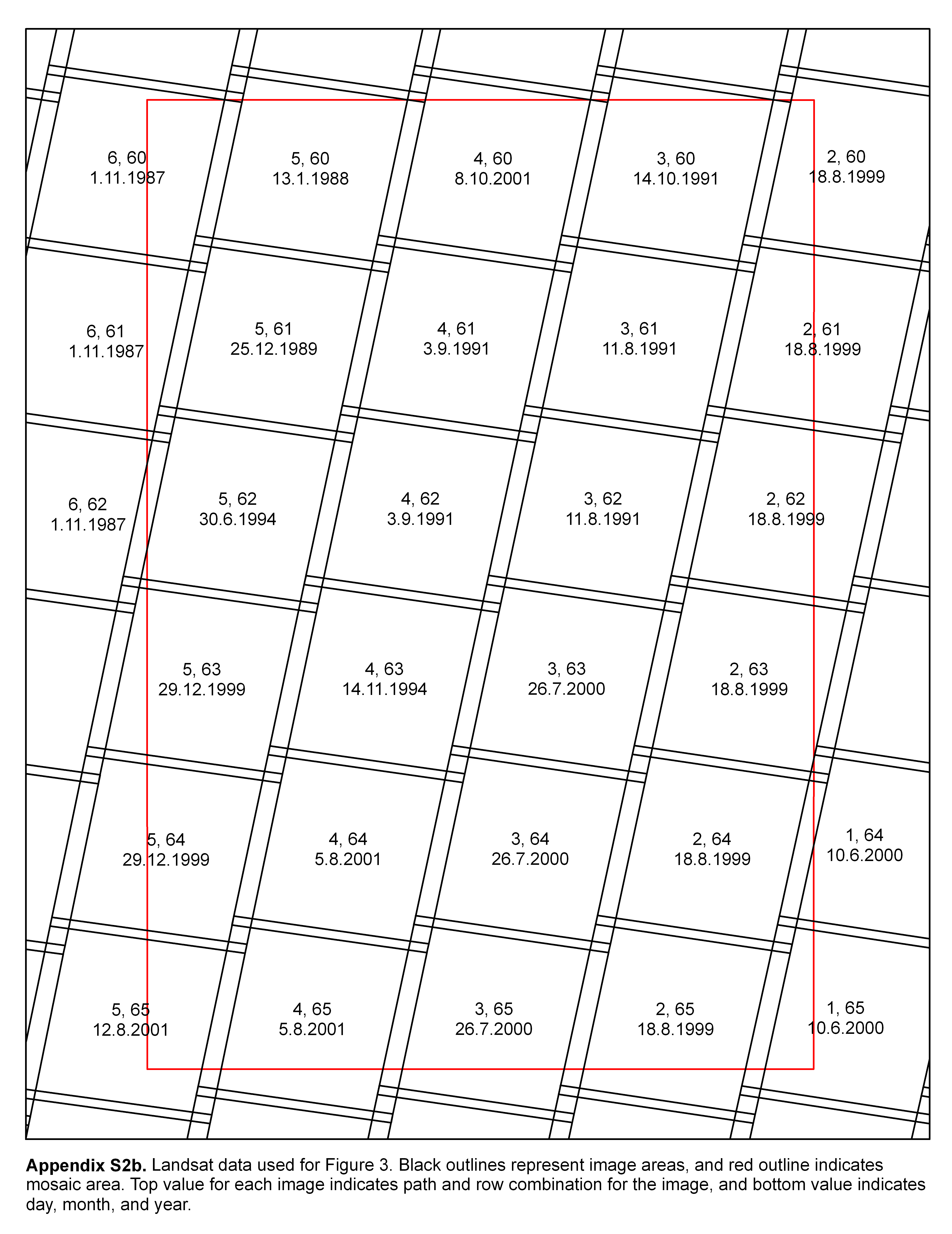


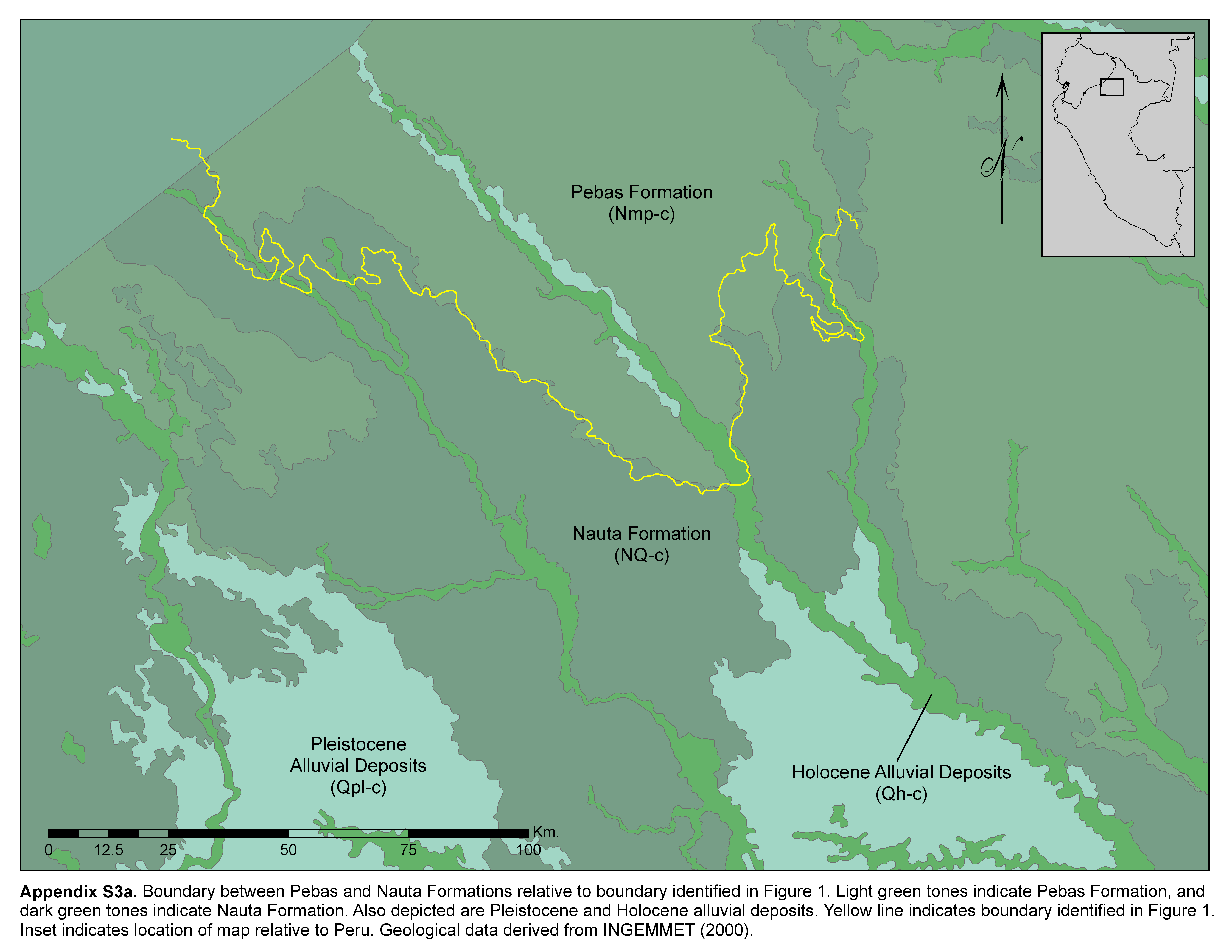


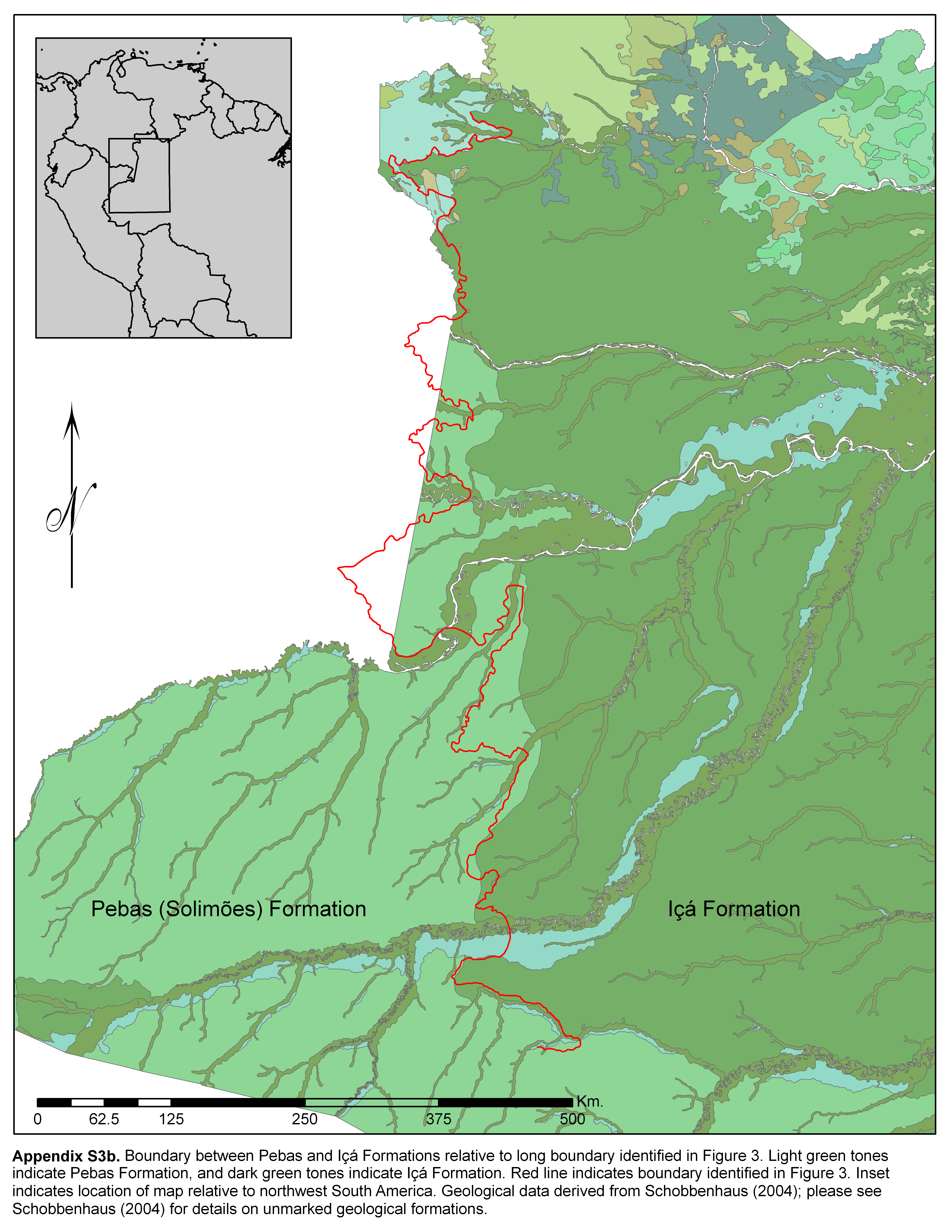


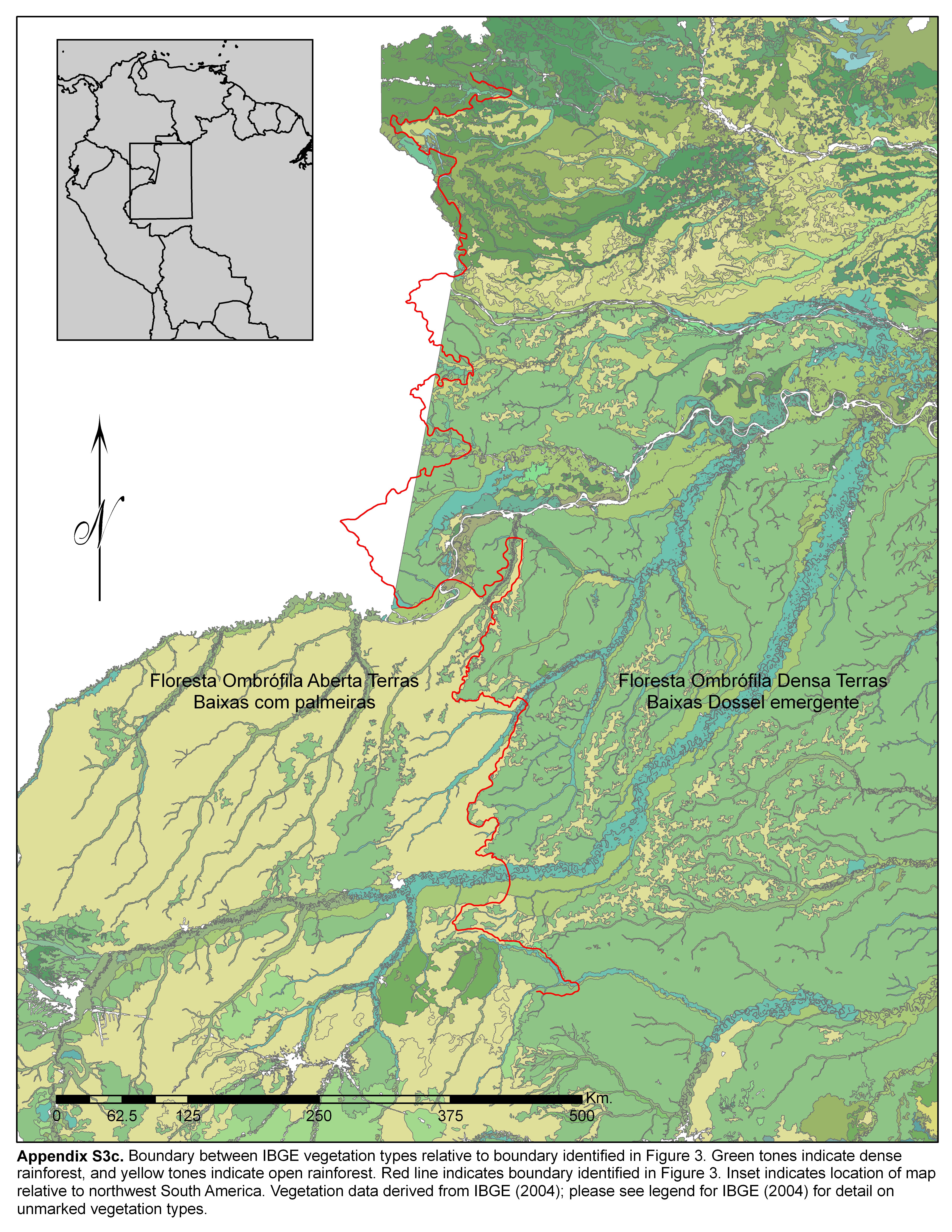

Supplement: Supplementary file 1 [file jbi0038-2136-SD1.doc]
