# Supplementary material for: Geological control of floristic composition in Amazonian forests
Source: J Biogeogr. 2011 Nov;38(11):2136–49. doi: 10.1111/j.1365-2699.2011.02585.x (PMC3253337; doi:10.1111/j.1365-2699.2011.02585.x)
Supplement: Supplementary file 2 [file jbi0038-2136-SD2.doc]

| **Appendix S4a. Indicator species analysis results for pteridophytes.** Results are given for analyses using either clustering-defined groups (sites divided into Nauta or Pebas Formation); cation-defined groups (sites with < or > 2.09 cmol(+)/kg sum of cations); or cation-defined groups with the richest quarter of transects removed. For each analysis, we report the group with which the species had the strongest association (i.e. the geological formation or cation-defined group), the indicator value (IV) for that association, and its significance (P). The table is sorted by geological group and then by species, and a value of "-" indicates that the corresponding species was not sufficiently frequent for the corresponding analysis. | | | | | | | | | |
| --- | --- | --- | --- | --- | --- | --- | --- | --- | --- |
|  |  |  |  |  |  |  |  |  |  |
|  | **Clustering-defined groups** | | | **Cation-defined groups, all transects** | | | **Cation-defined groups, excluding richest quarter of transects** | | |
| **Species** | **Geological formation^1^** | **IV^2^** | **P^3^** | **Cation group^4^** | **IV^2^** | **P^3^** | **Cation group^4^** | **IV^2^** | **P^3^** |
| *Adiantum amazonicum* A.R. Smith | Nauta | 18.82 | *** | Poor | 16.35 | ** | Poor | 14.08 | NS |
| *Adiantum cayennense* Willd. ex Klotzsch or *tuomistoanum* J. Prado | Nauta | 65.96 | *** | Poor | 60.78 | *** | Poor | 63.02 | *** |
| *Adiantum* sp. 12 | Nauta | 12.94 | ** | Poor | 12.79 | ** | Poor | 11.36 | NS |
| *Adiantum terminatum* Kunze ex Miq. | Nauta | 70.66 | *** | Poor | 67.61 | *** | Poor | 69.07 | *** |
| *Adiantum tomentosum* Kl. | Nauta | 72.96 | *** | Poor | 74.42 | *** | Poor | 76.25 | *** |
| *Asplenium cuneatum* Lam. | Nauta | 5.76 | NS | Poor | 4.19 | NS | - | - | - |
| *Asplenium hallii* Hook. | Nauta | 37.65 | *** | Poor | 37.21 | *** | Poor | 35 | *** |
| *Cnemidaria ewanii* (Alston) Tryon | Nauta | 33.46 | *** | Poor | 29.07 | ** | Poor | 29.2 | NS |
| *Cyathea lasiosora* (Mett. ex Kuhn) Domin | Nauta | 83.85 | *** | Poor | 82.88 | *** | Poor | 82.99 | *** |
| *Cyathea macrosora* (Baker) Domin | Nauta | 44.71 | *** | Poor | 44.19 | *** | Poor | 38.79 | *** |
| *Cyathea* sp. 5 | Nauta | 13.93 | NS | Poor | 8.81 | NS | Rich | 12.48 | NS |
| *Cyclodium meniscioides* (Willd.) Presl | Nauta | 86.73 | *** | Poor | 85.72 | *** | Poor | 85.95 | *** |
| *Cyclodium trianae* (Mett.) A.R.Smith | Nauta | 13.03 | * | Poor | 7.25 | NS | Rich | 7.12 | NS |
| *Danaea cartilaginea* Christenhusz & Tuomisto | Nauta | 52.97 | *** | Poor | 47.9 | *** | Poor | 49.18 | *** |
| *Danaea leprieurii* Kunze, bipinnata Tuomisto or sp.4 | Nauta | 55.39 | *** | Poor | 52.53 | *** | Poor | 54.14 | *** |
| *Danaea* sp. 24 | Nauta | 17.89 | ** | Poor | 19.83 | *** | Poor | 21.32 | * |
| *Elaphoglossum flaccidum* (Fée) Moore | Nauta | 24.27 | NS | Poor | 25.73 | * | Poor | 28 | NS |
| *Elaphoglossum luridum* (Fée) Christ | Nauta | 24.2 | ** | Poor | 23.92 | ** | Poor | 23.27 | NS |
| *Lindsaea bolivarensis* V. Marcano | Nauta | 14.12 | ** | Poor | 13.95 | ** | Poor | 13.75 | * |
| *Lindsaea divaricata* Kl. var. 1 | Nauta | 12.94 | ** | Poor | 12.79 | ** | - | - | - |
| *Lindsaea divaricata* Kl. var. 2 | Nauta | 39.28 | *** | Poor | 40.95 | *** | Poor | 44.02 | ** |
| *Lindsaea falcata* Dryand. | Nauta | 34.12 | *** | Poor | 33.72 | *** | Poor | 30 | *** |
| *Lindsaea guianensis* (Aubl.) Dryand. | Nauta | 43.53 | *** | Poor | 43.02 | *** | Poor | 42.5 | *** |
| *Lindsaea lancea* (L.) Bedd. var. lancea | Nauta | 55.29 | *** | Poor | 54.65 | *** | Poor | 51.28 | *** |
| *Lindsaea* sp. 18 | Nauta | 17.72 | ** | Poor | 11.56 | NS | Poor | 10.59 | NS |
| *Lindsaea* sp. 8 | Nauta | 55.32 | *** | Poor | 52.42 | *** | Poor | 53.98 | *** |
| *Lindsaea* sp. 9 | Nauta | 7.84 | NS | Poor | 9.38 | NS | Poor | 8.93 | NS |
| *Lindsaea taeniata* K.U. Kramer | Nauta | 47.17 | *** | Poor | 46.62 | *** | Poor | 47.76 | *** |
| *Lomariopsis nigropaleata* Holttum | Nauta | 63.79 | *** | Poor | 66.5 | *** | Poor | 73.6 | ** |
| *Lomariopsis prieuriana* Fée | Nauta | 23.58 | *** | Poor | 17.12 | * | Poor | 16.42 | NS |
| *Metaxya rostrata* (HBK.) Presl | Nauta | 56.67 | *** | Poor | 53.83 | *** | Poor | 55.38 | *** |
| *Microgramma megalophylla* (Desv.) Sota | Nauta | 16.47 | ** | Poor | 16.28 | ** | Poor | 15 | * |
| *Nephrolepis rivularis* (Vahl.) Mett. ex Krug | Nauta | 52.95 | ** | Poor | 53.99 | ** | Poor | 57.25 | NS |
| *Polybotrya pubens* Mart. | Nauta | 75.45 | *** | Poor | 70.18 | *** | Poor | 70.66 | *** |
| *Polybotrya sessilisora* R.C.Moran | Nauta | 69.41 | *** | Poor | 68.6 | *** | Poor | 67.5 | *** |
| *Polypodium adnatum* Klotzsch or dasypleuron Kze. | Nauta | 16.33 | NS | Poor | 17.89 | NS | Poor | 20.83 | NS |
| *Polytaenium guayanense* (Hieron.) Alston | Nauta | 11.29 | NS | Poor | 11.16 | NS | - | - | - |
| *Saccoloma elegans* Kaulfuss | Nauta | 8.24 | ** | - | - | - | - | - | - |
| *Saccoloma inaequale* (Kunze) Mettenius | Nauta | 74.36 | *** | Poor | 77.07 | *** | Poor | 81.63 | *** |
| *Salpichlaena hookeriana* (O. Kuntze) Alston | Nauta | 15.37 | ** | Poor | 15.19 | * | Poor | 16.33 | NS |
| *Salpichlaena volubilis* (Kaulf.) J. Smith | Nauta | 52.71 | *** | Poor | 59.8 | *** | Poor | 65.93 | *** |
| *Schizaea elegans* (Vahl.) Sw. | Nauta | 16.47 | *** | Poor | 16.28 | ** | Poor | 15 | * |
| *Selaginella lechleri* Hieron. | Nauta | 39.1 | *** | Poor | 40.82 | *** | Poor | 42.63 | *** |
| *Selaginella palmiformis* Alston ex Crabbe & Jermy | Nauta | 8.24 | * | - | - | - | - | - | - |
| *Selaginella parkeri* (Hook. & Grev.) Spring or sp. 8 | Nauta | 55.39 | *** | Poor | 52.53 | *** | Poor | 52.73 | *** |
| *Tectaria brauniana* (Karst.) C. Chr. | Nauta | 11.76 | ** | Poor | 11.63 | ** | - | - | - |
| *Trichomanes accedens* Presl | Nauta | 13.03 | * | Poor | 12.88 | * | Poor | 11.36 | NS |
| *Trichomanes cellulosum* Kl. | Nauta | 9.41 | * | Poor | 9.3 | * | - | - | - |
| *Trichomanes elegans* Rich. | Nauta | 80.07 | *** | Poor | 79.13 | *** | Poor | 78.82 | *** |
| *Trichomanes hostmannianum* (Kl.) Kunze | Nauta | 8.24 | * | - | - | - | - | - | - |
| *Trichomanes martiusii* Presl | Nauta | 9.41 | * | Poor | 9.3 | * | - | - | - |
| *Trichomanes pinnatum* Hedwig or sp. 1 or sp. 4 | Nauta | 81.73 | *** | Poor | 82.69 | *** | Poor | 82.47 | *** |
| *Trichomanes* sp. 6 (aff. elegans) | Nauta | 15.37 | ** | Poor | 17.44 | *** | Poor | 18.75 | * |
| *Trichomanes trollii* Bergdolt | Nauta | 47.09 | *** | Poor | 48.84 | *** | Poor | 48.78 | *** |
| *Triplophyllum dicksonioides* (Fée) Holttum or *funestum* (Kze.) Holttum or sp. 1 or sp. 2 | Nauta | 71.01 | *** | Poor | 70.18 | *** | Poor | 72.96 | *** |
| *Adiantum humile* Kunze or obliquum Willd. | Pebas | 51.48 | *** | Rich | 50.35 | ** | Rich | 38.52 | NS |
| *Adiantum pulverulentum* L. | Pebas | 86.83 | *** | Rich | 88.5 | *** | Rich | 74.24 | *** |
| *Anetium citrifolium* (L.) Splitgb. | Pebas | 60.99 | *** | Rich | 50.63 | *** | Rich | 27.78 | * |
| *Asplenium auritum* Sw. | Pebas | 33.96 | *** | Rich | 30.88 | *** | - | - | - |
| *Asplenium cirrhatum* Willd. | Pebas | 36.85 | *** | Rich | 29.09 | *** | Rich | 24.24 | * |
| *Asplenium delitescens* (Max.) A.R. Smith | Pebas | 15.09 | *** | Rich | 15.38 | *** | - | - | - |
| *Asplenium pearcei* Baker | Pebas | 79.6 | *** | Rich | 67.35 | *** | Rich | 41.22 | *** |
| *Asplenium serratum* L. or stuebelianum Hieron. | Pebas | 34.85 | NS | Poor | 34.55 | NS | Poor | 44.14 | NS |
| *Bolbitis lindigii* (Mett.) C.Chr. | Pebas | 83.36 | *** | Rich | 88.62 | *** | Rich | 74.69 | *** |
| *Bolbitis nicotianifolia* (Sw.) Alston | Pebas | 45.36 | *** | Rich | 46.23 | *** | Rich | 37.35 | *** |
| *Campyloneurum aphanophlebium* (Kunze) Moore | Pebas | 18.87 | *** | Rich | 15.58 | *** | - | - | - |
| *Campyloneurum fuscosquamatum* Lellinger | Pebas | 60.55 | *** | Rich | 55.38 | *** | Rich | 31.56 | *** |
| *Campyloneurum phyllitidis* (L.) Presl | Pebas | 12.94 | NS | Rich | 11.08 | NS | Rich | 8.89 | NS |
| *Campyloneurum* sp. 4 | Pebas | 21.75 | *** | Rich | 28.96 | *** | - | - | - |
| *Cyathea amazonica* R.C. Moran | Pebas | 13.21 | ** | - | - | - | - | - | - |
| *Cyathea bradei* (Windish) Lellinger or *pungens* (Willd.) Domin | Pebas | 48.45 | *** | Rich | 44.9 | ** | Rich | 33.82 | NS |
| *Cyathea cuspidata* Kunze | Pebas | 87.96 | *** | Rich | 82.89 | *** | Rich | 66.67 | *** |
| *Danaea acuminata* Tuomisto & R.C.Moran or *oblanceolata* Stolze | Pebas | 8.63 | NS | Rich | 6.73 | NS | Rich | 10.26 | NS |
| *Danaea nodosa* (L.) J. E. Smith | Pebas | 85.03 | *** | Rich | 83.37 | *** | Rich | 68.08 | *** |
| *Didymochlaena truncatula* (Sw.) J. Smith | Pebas | 87.63 | *** | Rich | 79.12 | *** | Rich | 65.31 | *** |
| *Diplazium grandifolium* (Sw.)Sw. var. andicola Stolze | Pebas | 56.6 | *** | Rich | 43.33 | *** | Rich | 36.82 | *** |
| *Diplazium pinnatifidum* Kunze | Pebas | 5.9 | NS | Rich | 8.65 | * | - | - | - |
| *Diplazium striatum* (L.) Presl | Pebas | 26.42 | ** | Rich | 23.21 | *** | - | - | - |
| *Elaphoglossum nigrescens* or sp. 11 | Pebas | 10.98 | * | Rich | 8.57 | NS | - | - | - |
| *Elaphoglossum raywaense* (Jenm.) Alston | Pebas | 19.85 | NS | Rich | 22.12 | NS | Rich | 24.95 | NS |
| *Lindsaea phassa* K.U. Kramer | Pebas | 23.58 | *** | Rich | 20.94 | *** | - | - | - |
| *Lomagramma guianensis* (Aubl.) Ching | Pebas | 26.03 | NS | Rich | 22.61 | NS | Rich | 26.11 | NS |
| *Lomariopsis fendleri* D.C.Eaton | Pebas | 62.47 | *** | Rich | 67.36 | *** | Rich | 48.4 | *** |
| *Lomariopsis japurensis* (Martius) J. Smith | Pebas | 87.96 | *** | Rich | 86.24 | *** | Rich | 72.34 | *** |
| *Lomariopsis latipinna* Stolze | Pebas | 39.66 | *** | Rich | 42.83 | *** | Rich | 40.83 | *** |
| *Microgramma fuscopunctata* (Hooker) Vareschi or *persicariifolia* (Schrader) Presl or *thurnii* (Baker) Tryon & Stolze | Pebas | 59.13 | *** | Rich | 53.38 | *** | Rich | 39.23 | NS |
| *Microgramma percussa* (Cav.) Sota | Pebas | 12.08 | ** | Rich | 15.58 | ** | - | - | - |
| *Pecluma hygrometrica* (Splitg.) Price or *ptilodon* (Kunze) Price | Pebas | 39.7 | *** | Rich | 40.47 | *** | - | - | - |
| *Polybotrya caudata* Kze. | Pebas | 47.42 | *** | Rich | 45.53 | *** | Rich | 32.69 | ** |
| *Polybotrya crassirhizoma* Lellinger | Pebas | 79.72 | *** | Rich | 81.25 | *** | Rich | 71.05 | *** |
| *Polybotrya fractiserialis* (Baker) J.Sm. | Pebas | 13.42 | ** | Rich | 13.68 | ** | - | - | - |
| *Polybotrya osmundacea* Willd. | Pebas | 56.41 | *** | Rich | 55.26 | *** | Rich | 42.86 | NS |
| *Polypodium caceresii* Sodiro | Pebas | 18.76 | *** | Rich | 19.12 | *** | Rich | 16.5 | ** |
| *Polytaenium cajenense* (Desv.) Benedict | Pebas | 56.66 | *** | Rich | 43.81 | *** | Rich | 31.37 | *** |
| *Pteris altissima* Poiret | Pebas | 15.09 | *** | Rich | 15.38 | *** | - | - | - |
| *Pteris pungens* Willd. | Pebas | 15.28 | *** | Rich | 19.23 | *** | - | - | - |
| *Selaginella exaltata* (Kunze) Spring | Pebas | 16.66 | NS | Rich | 21.49 | ** | Rich | 27.21 | ** |
| *Selaginella haematodes* (Kunze) Spring | Pebas | 26.53 | *** | Rich | 23.56 | *** | - | - | - |
| *Selaginella speciosa* A. Br. or sp.11 | Pebas | 8.04 | NS | Rich | 8.2 | NS | Rich | 11.34 | NS |
| *Stigmatopteris heterophlebia* (Baker) R.C. Moran or *opaca* (Baker) C.Chr. | Pebas | 26.53 | *** | Rich | 20.31 | *** | - | - | - |
| *Tectaria antioquoiana* (Baker) C.Chr. or *draconoptera* (D.C.Eaton) Copel. or *incisa* Cav. f. vivipara (Jenm.) Morton or sp. 2 | Pebas | 81.79 | *** | Rich | 86.67 | *** | Rich | 73.64 | *** |
| *Tectaria pilosa* (Fée) R.C.Moran or sp. 4 | Pebas | 66.04 | ** | Rich | 67.31 | *** | Rich | 44.44 | *** |
| *Thelypteris abrupta* (Desv.) Proctor | Pebas | 34.31 | *** | Rich | 34.97 | *** | Rich | 27.27 | *** |
| *Thelypteris ancyriothrix* (Rosenst.) A.R.Sm. | Pebas | 18.87 | *** | Rich | 19.23 | *** | - | - | - |
| *Thelypteris arcanum* (Maxon & Morton) Morton or *chrysodioides* (Fée) Morton | Pebas | 16.31 | ** | Rich | 13.74 | * | - | - | - |
| *Thelypteris biformata* (Rosenst.) Tryon | Pebas | 62.47 | *** | Rich | 67.36 | *** | Rich | 48.4 | *** |
| *Thelypteris glandulosa* (Desv.) Proctor var. *brachyodus* (Kunze) A.R.Sm. or *pennellii* A.R.Sm. | Pebas | 19.93 | ** | Rich | 30.77 | *** | - | - | - |
| *Thelypteris lugubriformis* (Rosenst.) R.Tryon | Pebas | 39.62 | *** | Rich | 40.38 | *** | - | - | - |
| *Thelypteris macrophylla* (Kunze) Morton | Pebas | 28.69 | *** | Rich | 23.94 | ** | Rich | 37.93 | *** |
| *Thelypteris opulenta* (Kaulf.) Fosberg | Pebas | 35.65 | *** | Rich | 33.24 | *** | Rich | 20 | *** |
| *Thelypteris pennata* (Poiret) Morton | Pebas | 49.06 | *** | Rich | 50 | *** | Rich | 40.74 | *** |
| *Thelypteris* sp. 1 | Pebas | 15.09 | *** | Rich | 15.38 | *** | - | - | - |
| *Trichomanes collariatum* Bosch | Pebas | 37.74 | *** | Rich | 38.46 | *** | - | - | - |
| *Trichomanes diversifrons* (Bory) Mett. ex Sadeb. | Pebas | 36.9 | *** | Rich | 37.61 | *** | Rich | 35.68 | *** |
|  |  |  |  |  |  |  |  |  |  |
| ^1^ Group of plots for which the highest IV (indicator value) was obtained: "Nauta" indicates Nauta Formation group; "Pebas" indicates Pebas Formation group | | | | | | | | | |
| ^2^ Indicator value | | | | | | | | | |
| ^3^ Statistical significance of indicator value (IV): NS = *P* > 0.05; * = *P* < 0.05; ** = *P* < 0.01; *** = *P* < 0.001 | | | | | | | | | |
| ^4^ Group of plots for which the highest IV (indicator value) was obtained: "Rich" indicates group with > 2.09 cmol(+)/kg sum of cations; "Poor" indicates group with < 2.09 cmol(+)/kg sum of cations | | | | | | | | | |

| **Appendix S4b. Indicator species analysis results for Melastomatacae species.** Results are given for analyses using either clustering-defined groups (sites divided into Nauta or Pebas Formation); cation-defined groups (sites with < or > 2.09 cmol(+)/kg sum of cations); or cation-defined groups with the richest quarter of transects removed. For each analysis, we report the group with which the species had the strongest association (i.e. the geological formation or cation-defined group), the indicator value (IV) for that association, and its significance (P). The table is sorted by geological group and then by species, and a value of "-" indicates that the corresponding species was not sufficiently frequent for the corresponding analysis. | | | | | | | | | |
| --- | --- | --- | --- | --- | --- | --- | --- | --- | --- |
|  |  |  |  |  |  |  |  |  |  |
|  | **Clustering-defined groups** | | | **Cation-defined groups, all transects** | | | **Cation-defined groups, excluding richest quarter of transects** | | |
| **Species** | **Geological group^1^** | **IV^2^** | **P^3^** | **Cation group^4^** | **IV^2^** | **P^3^** | **Cation group^4^** | **IV^2^** | **P^3^** |
| *Adelobotrys adscendens* (Sw.) Tr. | Nauta | 29.48 | *** | Poor | 29.48 | *** | Poor | 30.72 | * |
| *Adelobotrys marginata* Brade | Nauta | 44.16 | *** | Poor | 44.16 | *** | Poor | 50 | *** |
| *Adelobotrys praetexta* Pilg. | Nauta | 17.65 | * | Poor | 17.65 | ** | Poor | 16.13 | NS |
| *Adelobotrys rotundifolia* Triana | Nauta | 13.81 | NS | Poor | 13.81 | NS | Poor | 18.6 | NS |
| *Adelobotrys* sp. 7 | Nauta | 16.18 | * | Poor | 16.18 | ** | Poor | 17.74 | * |
| *Bellucia pentamera* Naud. | Nauta | 19.85 | NS | Poor | 19.85 | NS | Poor | 18.15 | NS |
| *Bellucia* sp. 2 | Nauta | 7.56 | NS | - | - | - | - | - | - |
| *Bellucia* sp. 5 | Nauta | 30.88 | *** | Poor | 30.88 | *** | Poor | 29.03 | ** |
| *Blakea* sp. 6 | Nauta | 11.76 | * | Poor | 11.76 | * | - | - | - |
| *Clidemia allardii* Wurdack | Nauta | 29.66 | ** | Poor | 29.66 | ** | Poor | 24.26 | NS |
| *Clidemia epibaterium* DC. | Nauta | 19.12 | ** | Poor | 19.12 | ** | - | - | - |
| *Clidemia longifolia* Gleason | Nauta | 69.63 | *** | Poor | 69.63 | *** | Poor | 78.47 | ** |
| *Clidemia piperifolia* Gleason | Nauta | 41.18 | *** | Poor | 41.18 | *** | Poor | 43.55 | *** |
| *Clidemia* sp. 10 | Nauta | 16.18 | * | Poor | 16.18 | ** | - | - | - |
| *Clidemia* sp. 2 | Nauta | 46.19 | *** | Poor | 46.19 | *** | Poor | 48.79 | ** |
| *Graffenrieda* sp. 2 | Nauta | 42.83 | *** | Poor | 42.83 | *** | Poor | 48.39 | *** |
| *Henriettella* sp. 3 | Nauta | 9.93 | NS | Poor | 9.93 | NS | Rich | 8 | NS |
| *Leandra aristigera* (Naud.) Cogn. | Nauta | 30.95 | *** | Poor | 30.95 | *** | Poor | 33.94 | ** |
| *Leandra candelabrum* (Macbr.) Wurdack | Nauta | 75.03 | *** | Poor | 75.03 | *** | Poor | 66.66 | *** |
| *Leandra glandulifera* (Tr.) Cogn. | Nauta | 38.24 | *** | Poor | 38.24 | *** | Poor | 35.48 | ** |
| *Leandra macdanielii* Wurdack | Nauta | 18.96 | NS | Poor | 18.96 | NS | Poor | 21.57 | NS |
| *Leandra secunda* (Don) Cogn. | Nauta | 16.37 | NS | Poor | 16.37 | NS | Poor | 20.65 | NS |
| *Leandra* sp. 4 | Nauta | 10.29 | NS | - | - | - | - | - | - |
| *Loreya* sp. 2 | Nauta | 22.06 | ** | Poor | 22.06 | ** | Poor | 24.19 | ** |
| *Maieta guianensis* Aubl. | Nauta | 77.07 | *** | Poor | 77.07 | *** | Poor | 77.44 | *** |
| *Maieta poeppigii* Mart. ex Cogn. | Nauta | 17 | NS | Poor | 17 | NS | Poor | 18.77 | NS |
| *Miconia abbreviata* Markgraf | Nauta | 11.76 | NS | Poor | 11.76 | NS | - | - | - |
| *Miconia ampla* Tr. | Nauta | 19.22 | * | Poor | 19.22 | ** | Poor | 21.08 | NS |
| *Miconia barbinervis* (Benth.) Tr. | Nauta | 25.08 | ** | Poor | 25.08 | ** | Poor | 27.5 | * |
| *Miconia carassana* Cogn. | Nauta | 77.94 | *** | Poor | 77.94 | *** | Poor | 77.45 | *** |
| *Miconia centrodesma* Naud. | Nauta | 52.29 | *** | Poor | 52.29 | *** | Poor | 55.76 | ** |
| *Miconia crassinervia* Cogn. | Nauta | 48.57 | *** | Poor | 48.57 | *** | Poor | 53.23 | *** |
| *Miconia dolichorrhyncha* Naud. | Nauta | 41.18 | *** | Poor | 41.18 | *** | Poor | 37.16 | ** |
| *Miconia egensis* Cogn. | Nauta | 11.76 | * | Poor | 11.76 | * | - | - | - |
| *Miconia elata* (Sw.) DC. | Nauta | 43.06 | NS | Poor | 43.06 | NS | Poor | 48.89 | NS |
| *Miconia fosteri* Wurdack | Nauta | 20.68 | ** | Poor | 20.68 | ** | Poor | 24.19 | * |
| *Miconia lourteigiana* Wurdack | Nauta | 12.25 | NS | Poor | 12.25 | NS | Poor | 16.26 | NS |
| *Miconia minutiflora* (Bonpl.) DC. | Nauta | 36.82 | ** | Poor | 36.82 | *** | Poor | 35.73 | * |
| *Miconia multispicata* Naud. | Nauta | 50 | *** | Poor | 50 | *** | Poor | 48.39 | *** |
| *Miconia nervosa* (Smith) Tr. | Nauta | 30.8 | NS | Poor | 30.8 | NS | Poor | 35.23 | NS |
| *Miconia paleacea* Cogn. | Nauta | 30.12 | NS | Poor | 30.12 | * | Poor | 33.18 | NS |
| *Miconia phanerostila* Pilger | Nauta | 32.35 | *** | Poor | 32.35 | *** | Poor | 24.29 | * |
| *Miconia pilgeriana* Ule | Nauta | 29.48 | ** | Poor | 29.48 | *** | Poor | 29.11 | * |
| *Miconia poeppigii* Triana | Nauta | 15.13 | NS | Poor | 15.13 | NS | Poor | 13.44 | NS |
| *Miconia prasina* (Sw.) DC. | Nauta | 44.32 | *** | Poor | 44.32 | ** | Poor | 45.04 | NS |
| *Miconia pterocaulon* Tr. | Nauta | 36.76 | *** | Poor | 36.76 | *** | Poor | 37.1 | *** |
| *Miconia pujana* Markgr. in Diels | Nauta | 32.35 | *** | Poor | 32.35 | *** | Poor | 27.42 | ** |
| *Miconia punctata* (Desr.) D. Don | Nauta | 33.36 | NS | Poor | 33.36 | NS | Poor | 35.41 | NS |
| *Miconia rimachii* Wurdack | Nauta | 25 | *** | Poor | 25 | ** | Poor | 24.19 | ** |
| *Miconia schunkei* Wurdack | Nauta | 48.53 | *** | Poor | 48.53 | *** | Poor | 46.97 | ** |
| *Miconia* sp. 105 | Nauta | 14.83 | * | Poor | 14.83 | * | Poor | 17.74 | * |
| *Miconia* sp. 11 | Nauta | 55.88 | *** | Poor | 55.88 | *** | Poor | 50.05 | *** |
| *Miconia* sp. 16 | Nauta | 20.72 | NS | Poor | 20.72 | NS | Poor | 23.31 | NS |
| *Miconia* sp. 18 | Nauta | 42.35 | ** | Poor | 42.35 | ** | Poor | 40.28 | NS |
| *Miconia* sp. 24 | Nauta | 8.56 | NS | Poor | 8.56 | NS | Poor | 10.32 | NS |
| *Miconia* sp. 35 | Nauta | 31.71 | NS | Poor | 31.71 | NS | Poor | 44.29 | * |
| *Miconia* sp. 46 | Nauta | 13.37 | NS | Poor | 13.37 | NS | Poor | 11.88 | NS |
| *Miconia* sp. 48 | Nauta | 14.71 | * | Poor | 14.71 | * | Poor | 16.13 | NS |
| *Miconia* sp. 49 | Nauta | 13.24 | * | Poor | 13.24 | * | - | - | - |
| *Miconia* sp. 50 | Nauta | 19.46 | * | Poor | 19.46 | * | Poor | 21.35 | NS |
| *Miconia* sp. 56 | Nauta | 13.24 | * | Poor | 13.24 | * | - | - | - |
| *Miconia* sp. 6 | Nauta | 25 | *** | Poor | 25 | ** | Poor | 19.35 | * |
| *Miconia* sp. 82 | Nauta | 13.24 | * | Poor | 13.24 | * | - | - | - |
| *Miconia* sp. 83 | Nauta | 16.18 | * | Poor | 16.18 | * | Poor | 17.74 | * |
| *Miconia* sp. 86 | Nauta | 19.12 | ** | Poor | 19.12 | ** | Poor | 15.01 | NS |
| *Miconia* sp. 87 | Nauta | 11.76 | * | Poor | 11.76 | * | - | - | - |
| *Miconia* sp. 89 | Nauta | 30.88 | *** | Poor | 30.88 | *** | Poor | 32.26 | ** |
| *Miconia* sp. 98 | Nauta | 19.12 | ** | Poor | 19.12 | ** | - | - | - |
| *Miconia* sp. 99 | Nauta | 13.69 | NS | Poor | 13.69 | NS | Poor | 16.26 | NS |
| *Miconia spichigeri* Wurdack | Nauta | 17.65 | ** | Poor | 17.65 | ** | - | - | - |
| *Miconia splendens* (Sw.) Grieseb. | Nauta | 11.76 | * | Poor | 11.76 | NS | - | - | - |
| *Miconia subspicata* Wurdack | Nauta | 33 | NS | Poor | 33 | NS | Poor | 38.71 | NS |
| *Miconia tetragona* Cogn. | Nauta | 22.15 | ** | Poor | 22.15 | ** | Poor | 21.35 | NS |
| *Miconia tetrasperma* Gleason | Nauta | 44.16 | *** | Poor | 44.16 | *** | Poor | 43.76 | ** |
| *Miconia tomentosa* (L. C. Rich.) D. Don | Nauta | 69.18 | *** | Poor | 69.18 | *** | Poor | 76.64 | *** |
| *Miconia traillii* Cogn. | Nauta | 52.98 | *** | Poor | 52.98 | *** | Poor | 51.66 | *** |
| *Miconia umbriensis* Wurdack | Nauta | 39.71 | *** | Poor | 39.71 | *** | Poor | 32.82 | * |
| *Miconia zubenatana* Macbr. | Nauta | 8.01 | NS | Poor | 8.01 | NS | - | - | - |
| *Monolena primulaeflora* Hook. | Nauta | 7.56 | NS | - | - | - | - | - | - |
| *Ossaea araneifera* Mkgf. | Nauta | 75 | *** | Poor | 75 | *** | Poor | 71 | *** |
| *Ossaea boliviensis* (Cogn.) Gleason | Nauta | 34.81 | NS | Poor | 34.81 | NS | Poor | 42.38 | NS |
| *Ossaea bullifera* (Pilger) Gleason | Nauta | 19.12 | ** | Poor | 19.12 | ** | Poor | 15.01 | NS |
| *Ossaea cucullata* Gleason | Nauta | 54.72 | *** | Poor | 54.72 | *** | Poor | 64.55 | *** |
| *Tococa caryophyllea* (DC.) Renner | Nauta | 22.06 | ** | Poor | 22.06 | ** | Poor | 19.35 | * |
| *Tococa* sp. 2 | Nauta | 63.27 | *** | Poor | 63.27 | *** | Poor | 61.33 | *** |
| *Tococa ulei* Pilger | Nauta | 63.39 | *** | Poor | 63.39 | *** | Poor | 63.2 | ** |
| *Adelobotrys klugii* Wurdack | Pebas | 27.61 | *** | Rich | 27.61 | *** | Rich | 24.62 | ** |
| *Adelobotrys scandens* (Aubl.) DC. subsp. *elongata* Schulman | Pebas | 19.44 | ** | - | - | - | - | - | - |
| *Adelobotrys tessmannii* Markgr. subsp. latifolia Schulman | Pebas | 36.76 | *** | Rich | 36.76 | *** | - | - | - |
| *Blakea rosea* (R. & P.) D. Don | Pebas | 40.5 | * | Rich | 40.5 | * | Rich | 28.85 | NS |
| *Clidemia dimorphica* Macbr. | Pebas | 31.41 | NS | Rich | 31.41 | NS | Rich | 33.79 | NS |
| *Clidemia epiphytica* (Tr.) Cogn. | Pebas | 16.67 | NS | Rich | 16.67 | NS | Rich | 16 | NS |
| *Clidemia heterophylla* (Desr.) Gleason | Pebas | 32.11 | ** | Rich | 32.11 | *** | Rich | 37.89 | ** |
| *Clidemia septuplinervia* Cogn. | Pebas | 67.06 | *** | Rich | 67.06 | *** | Rich | 66.18 | *** |
| *Clidemia serpens* (Tr.) Cogn. | Pebas | 11.34 | NS | Rich | 11.34 | NS | - | - | - |
| *Clidemia* sp. 3 | Pebas | 25 | *** | Rich | 25 | *** | - | - | - |
| *Clidemia sprucei* Gleason | Pebas | 21.37 | ** | Rich | 21.37 | *** | - | - | - |
| *Henriettea* sp. 1 | Pebas | 8.68 | NS | Rich | 8.68 | NS | - | - | - |
| *Leandra caquetana* Sprague | Pebas | 30.77 | *** | Rich | 30.77 | *** | - | - | - |
| *Leandra longicoma* Cogn. | Pebas | 61.75 | *** | Rich | 61.75 | *** | Rich | 50.14 | *** |
| *Miconia acutipetala* Sprague | Pebas | 36.76 | *** | Rich | 36.76 | *** | Rich | 38.46 | *** |
| *Miconia aureoides* Cogn. | Pebas | 22.22 | *** | Rich | 22.22 | *** | - | - | - |
| *Miconia decurrens* Cogn. | Pebas | 18.15 | NS | Rich | 18.15 | NS | Rich | 16.88 | NS |
| *Miconia grandifolia* Ule | Pebas | 53.78 | *** | Rich | 53.78 | *** | Rich | 38.46 | *** |
| *Miconia lamprophylla* Tr. | Pebas | 36.3 | *** | Rich | 36.3 | *** | Rich | 41.67 | *** |
| *Miconia lugonis* Wurdack | Pebas | 24.01 | *** | Rich | 24.01 | *** | Rich | 38.46 | *** |
| *Miconia napoana* Wurdack | Pebas | 9.88 | NS | Rich | 9.88 | NS | Rich | 9.62 | NS |
| *Miconia procumbens* (Gleason) Wurdack | Pebas | 38.89 | *** | Rich | 38.89 | *** | - | - | - |
| *Miconia serrulata* (DC.) Naud. | Pebas | 48.31 | *** | Rich | 48.31 | *** | Rich | 51.43 | *** |
| *Miconia* sp. 108 | Pebas | 25 | *** | Rich | 25 | *** | - | - | - |
| *Miconia* sp. 15 | Pebas | 49 | *** | Rich | 49 | *** | Rich | 41.67 | *** |
| *Miconia* sp. 19 | Pebas | 26.08 | ** | Rich | 26.08 | ** | Rich | 22.86 | ** |
| *Miconia* sp. 27 | Pebas | 35.56 | *** | Rich | 35.56 | *** | Rich | 38.46 | *** |
| *Miconia* sp. 3 | Pebas | 40.37 | ** | Rich | 40.37 | ** | Rich | 35.16 | NS |
| *Miconia* sp. 33 | Pebas | 10 | NS | Rich | 10 | NS | - | - | - |
| *Miconia* sp. 37 | Pebas | 19.44 | ** | - | - | - | - | - | - |
| *Miconia* sp. 40 | Pebas | 28.41 | *** | Rich | 28.41 | *** | Rich | 16.33 | NS |
| *Miconia* sp. 43 | Pebas | 17.01 | ** | Rich | 17.01 | ** | - | - | - |
| *Miconia* sp. 44 | Pebas | 36.76 | *** | Rich | 36.76 | *** | Rich | 40.5 | *** |
| *Miconia* sp. 5 | Pebas | 9.92 | NS | - | - | - | - | - | - |
| *Miconia* sp. 60 | Pebas | 33.33 | *** | Rich | 33.33 | *** | - | - | - |
| *Miconia* sp. 73 | Pebas | 44.6 | *** | Rich | 44.6 | *** | Rich | 40.5 | *** |
| *Miconia spennerostachya* Naud. | Pebas | 25 | *** | Rich | 25 | *** | - | - | - |
| *Miconia trinervia* (Sw.) D. Don ex Loud. | Pebas | 11.11 | NS | Rich | 11.11 | NS | - | - | - |
| *Miconia triplinervis* R. & P. | Pebas | 33.33 | *** | Rich | 33.33 | *** | - | - | - |
| *Tococa caquetana* Sprague | Pebas | 57.67 | *** | Rich | 57.67 | *** | Rich | 46.51 | NS |
| *Triolena amazonica* (Pilger) Wurdack | Pebas | 80.65 | *** | Rich | 80.65 | *** | Rich | 48 | *** |
|  |  |  |  |  |  |  |  |  |  |
| ^1^ Group of plots for which the highest IV (indicator value) was obtained: "Nauta" indicates Nauta Formation group; "Pebas" indicates Pebas Formation group | | | | | | | | | |
| ^2^ Indicator value | | | | | | | | | |
| ^3^ Statistical significance of indicator value (IV): NS = *P* > 0.05; * = *P* < 0.05; ** = *P* < 0.01; *** = *P* < 0.001 | | | | | | | | | |
| ^4^ Group of plots for which the highest IV (indicator value) was obtained: "Rich" indicates group with > 2.09 cmol(+)/kg sum of cations; "Poor" indicates group with < 2.09 cmol(+)/kg sum of cations | | | | | | | | | |

| **Appendix S4c. Indicator species analysis results for tree species.** Results are reported for both high abundance species (at least 1 individual per hectare) and low abundance species (less than one individual per hectare), and were calculated for two cation-defined groups (sites with < or > 2.09 cmol(+)/kg sum of cations). For each analysis we report the group with which the species had the strongest association (i.e. "Rich" or "Poor"), the indicator value (IV) for that association, and its significance (P). The table is sorted by abundance group and then by species. | | | | |
| --- | --- | --- | --- | --- |
|  |  |  |  |  |
| **Species** | **Abundance^1^** | **Cation group^2^** | **IV^3^** | **P^4^** |
| *Astrocaryum chambira* Burret | High | Poor | 75.63 | * |
| *Astrocaryum murumuru* Mart. | High | Rich | 64.39 | NS |
| *Attalea racemosa* Spruce | High | Rich | 53.33 | NS |
| *Cordia nodosa* Lam. | High | Poor | 40.6 | NS |
| *Eschweilera coriacea* (DC.) S.A. Mori | High | Poor | 79.07 | ** |
| *Eschweilera tessmannii* R. Knuth | High | Poor | 28.57 | NS |
| *Guarea grandifolia* DC. | High | Rich | 67.67 | * |
| *Guarea kunthiana* A. Juss. | High | Rich | 69.39 | NS |
| *Iriartea deltoidea* Ruiz & Pav. | High | Rich | 85 | ** |
| *Iryanthera juruensis* Warb. | High | Poor | 36.57 | NS |
| *Iryanthera laevis* Markgr. | High | Poor | 36.73 | NS |
| *Iryanthera paraensis* Huber | High | Rich | 49.62 | NS |
| *Iryanthera ulei* Warb. | High | Poor | 40.82 | NS |
| *Leonia glycycarpa* Ruiz & Pav. | High | Poor | 33.77 | NS |
| *Lepidocaryum tenue* Mart. | High | Poor | 99.29 | *** |
| *Nealchornea yapurensis* Huber | High | Rich | 32.65 | NS |
| *Oenocarpus bataua* Mart. | High | Poor | 66.59 | * |
| *Otoba glycycarpa* (Ducke) W.A. Rodrigues & T.S. Jaramillo | High | Rich | 82.76 | * |
| *Otoba parvifolia* (Markgr.) A.H. Gentry | High | Rich | 89.29 | * |
| *Phytelephas macrocarpa* Ruiz & Pav. | High | Rich | 43.96 | NS |
| *Pouteria torta* (Mart.) Radlk. | High | Poor | 74.29 | * |
| *Protium nodulosum* Swart | High | Rich | 32.65 | NS |
| *Rinorea lindeniana* (Tul.) Kuntze | High | Poor | 28.57 | NS |
| *Rinorea racemosa* (Mart.) Kuntze | High | Poor | 85.71 | ** |
| *Senefeldera skutchiana* Croizat | High | Poor | 71.43 | * |
| *Tetrathylacium macrophyllum* Poepp. | High | Rich | 80 | ** |
| *Trymatococcus amazonicus* Poepp. & Endl. | High | Poor | 53.06 | NS |
| *Virola calophylla* (Spruce) Warb. | High | Poor | 85.11 | ** |
| *Virola pavonis* (A. DC.) A.C. Sm. | High | Poor | 71.43 | * |
| *Brosimum utile* (Kunth) Oken ex J. Presl | Low | Poor | 24.49 | NS |
| *Carpotroche longifolia* (Poepp.) Benth. | Low | Rich | 33.33 | NS |
| *Casearia fasciculata* (Ruiz & Pav.) Sleumer | Low | Poor | 21.43 | NS |
| *Conceveiba rhytidocarpa* Müll. Arg. | Low | Rich | 30.77 | NS |
| *Diclinanona tessmannii* Diels | Low | Poor | 71.43 | * |
| *Drypetes amazonica* Steyerm. | Low | Rich | 59.52 | NS |
| *Eschweilera gigantea* (R. Knuth) J.F. Macbr. | Low | Rich | 40.82 | NS |
| *Eschweilera rufifolia* S.A. Mori | Low | Poor | 35.16 | NS |
| *Euterpe precatoria* Mart. | Low | Poor | 38.1 | NS |
| *Guarea pterorachis* Harms | Low | Rich | 65.48 | * |
| *Hirtella racemosa* Lam. | Low | Poor | 52.38 | NS |
| *Inga alba* (Sw.) Willd. | Low | Rich | 50 | NS |
| *Inga pruriens* Poepp. | Low | Rich | 21.43 | NS |
| *Iryanthera macrophylla* (Benth.) Warb. | Low | Poor | 46.75 | NS |
| *Licania macrocarpa* Cuatrec. | Low | Poor | 25.71 | NS |
| *Macrolobium limbatum* Spruce ex Benth. | Low | Poor | 52.75 | NS |
| *Matisia bracteolosa* Ducke | Low | Poor | 25.71 | NS |
| *Minquartia guianensis* Aubl. | Low | Poor | 23.81 | NS |
| *Naucleopsis ulei* (Warb.) Ducke | Low | Rich | 28.57 | NS |
| *Ophiocaryon heterophyllum* (Benth.) Urb. | Low | Poor | 23.38 | NS |
| *Pachira insignis* (Sw.) Sw. ex Savigny | Low | Rich | 47.62 | NS |
| *Pourouma cecropiifolia* Mart. | Low | Poor | 27.27 | NS |
| *Pseudolmedia laevigata* Trécul | Low | Poor | 64.29 | * |
| *Pseudolmedia laevis* (Ruiz & Pav.) J.F. Macbr. | Low | Rich | 64.29 | NS |
| *Roucheria punctata* (Ducke) Ducke | Low | Poor | 71.43 | * |
| *Ryania speciosa* Vahl | Low | Rich | 50.79 | NS |
| *Siparuna cristata* (Poepp. & Endl.) A. DC. | Low | Rich | 32.14 | NS |
| *Socratea exorrhiza* (Mart.) H. Wendl. | Low | Poor | 45.71 | NS |
| *Sorocea hirtella* Mildbr. | Low | Rich | 51.95 | NS |
| *Tetragastris panamensis* (Engl.) Kuntze | Low | Rich | 43.96 | NS |
| *Tetrastylidium peruvianum* Sleumer | Low | Poor | 23.81 | NS |
| *Theobroma obovatum* Klotzsch ex Bernoulli | Low | Poor | 24.49 | NS |
| *Theobroma subincanum* Mart. | Low | Poor | 44.44 | NS |
| *Virola duckei* A.C. Sm. | Low | Rich | 30 | NS |
| *Virola multinervia* Ducke | Low | Rich | 21.43 | NS |
| *Virola peruviana* (A. DC.) Warb. | Low | Poor | 25 | NS |
|  |  |  |  |  |
| ^1^ Average abundance of species: "High" indicates ≥ 1 individual per plot; "Low" indicates < 1 individual per plot. | | | | |
| ^2^ Group of plots for which the highest IV (indicator value) was obtained: "Rich" indicates group with > 2.09 cmol(+)/kg sum of cations; "Poor" indicates group with < 2.09 cmol(+)/kg sum of cations | | | | |
| ^3^ Importance value | | | | |
| ^4^ Statistical significance of indicator value (IV): NS = *P* > 0.05; * = *P* < 0.05; ** = *P* < 0.01; *** = *P* < 0.001 | | | | |
